# Disentangling drivers of air pollutant and health risk changes during the COVID-19 lockdown in China

**DOI:** 10.1038/s41612-022-00276-0

**Published:** 2022-06-30

**Authors:** Fuzhen Shen, Michaela I. Hegglin, Yuanfei Luo, Yue Yuan, Bing Wang, Johannes Flemming, Junfeng Wang, Yunjiang Zhang, Mindong Chen, Qiang Yang, Xinlei Ge

**Affiliations:** 1 Jiangsu Key Laboratory of Atmospheric Environment Monitoring and Pollution Control, Collaborative Innovation Center of Atmospheric Environment and Equipment Technology, School of Environmental Science and Engineering, Nanjing University of Information Science and Technology, 210044 Nanjing, China; 2 Department of Meteorology, University of Reading, Reading, RG6 6BX UK; 3 Institute of Energy and Climate Research, IEK-7: Stratosphere, Forschungszentrum Jülich, 52425 Jülich, Germany; 4 4Paradigm Inc., 100000 Beijing, China; 5 Jining Meteorological Bureau, 272000 Shandong, China; 6 Henley Business School, University of Reading, Reading, RG6 6UD UK; 7 ECMWF, Shinfield Park, Reading, RG2 9AX UK; 8 John A. Paulson School of Engineering and Applied Sciences, Harvard University, Cambridge, MA 02138 USA; 9 Hongkong University of Science and Technology, 999007 Hong Kong, China

**Keywords:** Atmospheric chemistry, Environmental impact, Environmental monitoring

## Abstract

The COVID-19 restrictions in 2020 have led to distinct variations in NO_2_ and O_3_ concentrations in China. Here, the different drivers of anthropogenic emission changes, including the effects of the Chinese New Year (CNY), China’s 2018–2020 Clean Air Plan (CAP), and the COVID-19 lockdown and their impact on NO_2_ and O_3_ are isolated by using a combined model-measurement approach. In addition, the contribution of prevailing meteorological conditions to the concentration changes was evaluated by applying a machine-learning method. The resulting impact on the multi-pollutant Health-based Air Quality Index (HAQI) is quantified. The results show that the CNY reduces NO_2_ concentrations on average by 26.7% each year, while the COVID-lockdown measures have led to an additional 11.6% reduction in 2020, and the CAP over 2018–2020 to a reduction in NO_2_ by 15.7%. On the other hand, meteorological conditions from 23 January to March 7, 2020 led to increase in NO_2_ of 7.8%. Neglecting the CAP and meteorological drivers thus leads to an overestimate and underestimate of the effect of the COVID-lockdown on NO_2_ reductions, respectively. For O_3_ the opposite behavior is found, with changes of +23.3%, +21.0%, +4.9%, and −0.9% for CNY, COVID-lockdown, CAP, and meteorology effects, respectively. The total effects of these drivers show a drastic reduction in multi-air pollutant-related health risk across China, with meteorology affecting particularly the Northeast of China adversely. Importantly, the CAP’s contribution highlights the effectiveness of the Chinese government’s air-quality regulations on NO_2_ reduction.

## Introduction

Air pollution ranks as the 4th leading risk factor contributing to 6.67 million premature deaths globally in 2019, with 1.85 millions of these deaths recorded in China alone^1^. Poor air quality is driven by pollutant emissions of NOx, CO, and SO_2_, which lead to the production of secondary air pollutants like ozone (O_3_) and particulate matter (PM) and can be strongly modulated by the prevailing meteorological conditions^2,3^. Any variations in emissions, atmospheric chemistry processes, and meteorological conditions thus could impact the air quality in one region/city. To understand the relative roles of changes in the above factors on air quality and related health risks, the influence of these confounding factors must be isolated.

Starting on January 23, 2020, the Chinese government implemented different levels of lockdown restrictions in different regions/cities, one day before the Chinese New Year (CNY) in order to slow down the transmission of the novel coronavirus disease 2019 (COVID-19). During the CNY holiday, some primary air pollutants like nitrogen dioxide (NO_2_), generally show a decline due to the temporary suspension of economic activities and closure of factories in China^4–6^. While the COVID-19 lockdown coincided with the CNY holiday, it has extended and intensified the CNY restrictions on transportation and industrial activities^7,8^. Correspondingly, satellite observations of air pollutants by the National Aeronautics and Space Administration (NASA) and the European Space Agency (ESA) have revealed unusually stark decreases in NO_2_ across the whole of China until the relief of the lockdown measures^9^. Notably, several satellite-based observation studies have demonstrated that tropospheric NO_2_ concentrations showed declining trends in some regions of China already before 2020^10–12^, with ground-based observation studies however highlighting that these reductions were not significant across all regions^2,13,14^. These improvements in air quality were attributed to the implementation of China’s Clean Air Plan (CAP) from 2013 to 2017. In detail, the CAP measures include the reduction of coal-fired emissions, industrial emissions, vehicle emissions, dust emissions, and other measures^13^. To complete the target of CAP from 2013 to 2017, some local governments also carried out a series of supplementary control measures (referred to as the Comprehensive Action) whereas its primary target mainly focused on the reduction of PM rather than NO_2_^13^. Thereafter, to better tackle the issue of NOx pollution, China introduced a new 3-year action plan to combat air pollution from 2018 to 2020^15^, leading to yet more stringent control measures on NOx emissions. Thus, short-term policy measures (the COVID-19 lockdown restrictions) and holidays (the CNY) coupled with the two long-term CAPs in China have increased the anthropogenic impact on NO_2_ reductions. Except for these emission reductions, changes in meteorological conditions, especially in atmospheric transport and Planetary Boundary Layer (PBL) height, have played an important role not only in driving single air pollution events^16–18^ but also in determining increments in NO_2_ in about 70 cities in China during the COVID-19 lockdown^19^. Overall, these different anthropogenic emission drivers, combined with changing meteorological conditions, represent confounding factors and provide a challenge in the differentiation and attribution of air-quality changes during the COVID-19 lockdown.

After the outbreak of the COVID-19 pandemic in 2020, a large number of studies have attempted to quantify the effect of the lockdown measures on emission reductions in primary air pollutants using a wide range of evaluation approaches. When compared to the NO_2_ concentrations averaged over an equivalent time period to the COVID-lockdown and over several years before 2020 (Baseline-I) (note, with the total length of this time period differing among studies)^20–25^, the average percentage reduction in NO_2_ from ground-based measurements was found to be 51.5 ± 14.3%. When only compared to the NO_2_ concentration during the same period of 2019 as a reference (Baseline-II)^8,26–32^, the average percentage reduction in NO_2_ is 45.7 ± 15.8%. When considering the average percentage NO_2_ concentration before the COVID-19 lockdown in 2020 as a reference (Baseline-III)^19,33–47^, the NO_2_ average percentage reduction was 53 ± 12.7%. Meanwhile, results from satellite-based studies demonstrated that average percentage reductions in NO_2_ were 44.3 ± 21.0%, 37.7 ± 10.0%, and 53.4 ± 14.9% when the NO_2_ concentration comparisons used Baseline-I^5,48^, Baseline-II^49–52^, and Baseline-III^5,41,49,50^ as a reference, respectively. Yet other studies, quantifying the NO_2_ concentration changes under “Business As Usual” (hereafter referred to as BAU) emission strength and using chemistry transport model simulations^6,41,52–57^ or machine-learning (ML) methods^58^, found that the NO_2_ concentrations dropped by 54.4 ± 8.3% and 44.1 ± 9.4% during the COVID-19 lockdown period, respectively. However, there are some limitations to the above studies. Firstly, most of the studies do not quantify the impact of CNY and CAP on NOx emission reductions during the COVID-19 lockdown period. Furthermore, only few studies account for the potential effects of changes in the meteorological conditions on air pollutant concentrations during the COVID-19 period at a national level. For example, Song et al. applied a statistical model (referred to as the meteorology-pollution decomposition method) to differentiate meteorological and anthropogenic effects on PM_2.5_ across China^59^. Liu et al. used a Chemical Transport Model to investigate the impacts of meteorological changes and emission reductions on O_3_^60^. At last, most studies focused on the concentration change of just one or two air pollutants and only for a subset of cities in China, thus not reflecting the influence of the COVID-19 lockdown on the air quality and related health risks comprehensively.

The study presented here aims at a more detailed attribution of observed NO_2_ and O_3_ concentration changes to potential drivers at the city level across China during 2020, by quantifying the contributions of the CAP and the COVID-19 lockdown measures to the variation of NO_2_ and O_3_, and also by exploring the role of changes in meteorology to the observed NO_2_ and O_3_ concentration changes. To this end, we use a combined model-measurement approach, exploiting observations from in situ measurement stations, air-quality modeling data available from the European Copernicus Climate Service, and emulations based on ML. We finally quantify the changes in health risk resulting from varying air pollutant concentrations during the COVID-19 lockdown period, extending our evaluation method to six major air pollutants and offering results at the city level across China.

## Results and discussion

### Identifying different drivers of anthropogenic emission reductions

**Fig. 1:**
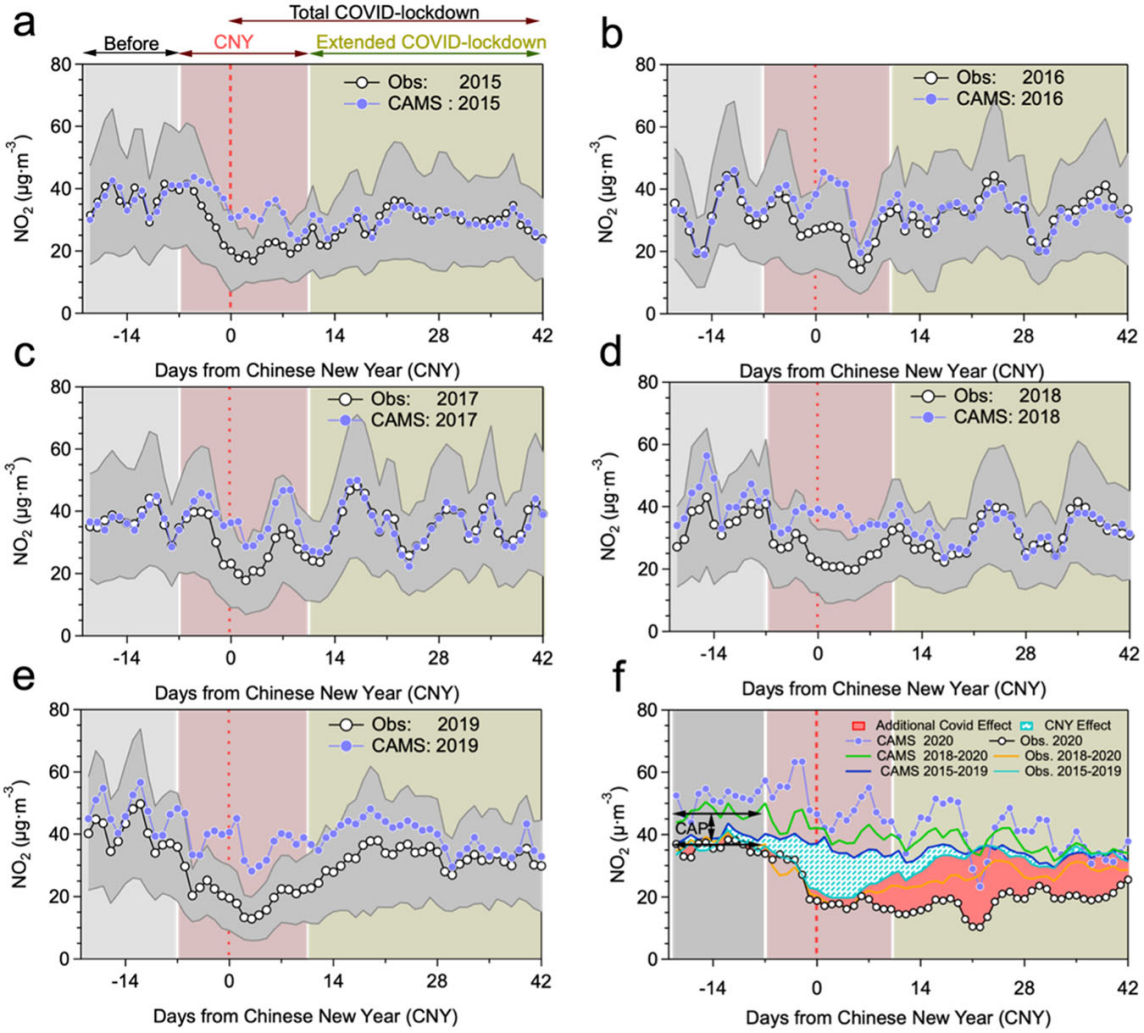
Comparison between observations and CAMSRA for NO_2_ across China. **a**–**e** show comparison results of the daily averaged NO_2_ from observations and CAMSRA during the equivalent time of the 2020 COVID-lockdown from 2015 to 2019. **f** shows the comparison result in 2020 and additionally the corresponding data for the time average over 2015 to 2019 (blue line) and 2018 to 2020 (green line). The solid purple circle represents the average daily NO_2_ concentration of CAMSRA, the black circle represents the average daily NO_2_ concentration of observation across China, respectively. The red dash line represents the Chinese New Year’s day (CNY). Different background colors represent different time periods including “Before CNY” (gray shaded area) ranged from days −21 to −8 from the CNY’s day, “CNY” (red shaded area) ranged from days −7 to +10 from the CNY’s day, “Extended COVID-lockdown” (moss shade area) ranged from days +11 to +42 from the CNY’s day, and “Total COVID-lockdown” ranges from days −1 to +42 from the CNY’s day. The gap between the two black arrow lines represents the China’s Clean Air Plan effect (CAP).

Figure 1 illustrates the evaluation of the CNY contribution to observed NO_2_ decreases. To better evaluate the anthropogenic emission reductions caused by the CNY festivities, different time periods including Before CNY, CNY, Extended COVID-lockdown, and Total COVID-lockdown are defined (see Supplementary Table 1 for definitions of time periods relative to the CNY day). Figure 1f shows the daily variation of the CAMSRA (Copernicus Atmosphere Monitoring Service Reanalysis) NO_2_ and the observed NO_2_ in 2020. It should be noted that CAMSRA can be used as counterfactual for a world in which the COVID-lockdown, CNY, and air-quality regulations did not happen because these emission reductions were not accounted for in the emissions database used. Although the observed NO_2_ and the CAMSRA NO_2_ show significantly larger differences during the COVID-19 lockdown period compared to Before CNY, it is not clear whether those decreases were fully attributable to the lockdown. We hence show the daily variations of NO_2_ concentrations not only in 2020 but also for the previous years 2015 to 2019 (Fig. 1a–e), to reveal the roles of other factors in the variations of NO_2_ concentrations in years without a COVID-19 lockdown. From 2015 to 2019, a difference between CAMSRA NO_2_ and observed NO_2_ concentrations always appears during the CNY period, highlighting that the CNY generally exhibits an anthropogenic emission reduction. Near perfect agreement between CAMSRA and observed NO_2_ is found for 2015–2017 during Before CNY and also after CNY. Unlike 2015–2017, however, CAMSRA NO_2_ did not match the observed NO_2_ quite that well before and after the CNY in 2018 and 2019, showing slight overestimates of the observed values. The reason for this behavior is that the first CAP had virtually no effect on NO_2_ from 2015 to 2017, but the new 3-year CAP from 2018 to 2020 (CAP_2018–2020_) led to a perceptible decline, especially in 2019. Some recent studies^2,14^, which had focused on the concentration changes of nationwide NO_2_, provide support for our interpretation that NO_2_ had no significant decline over the years 2015–2017. These studies also expected that this phenomenon would be improved from 2018 to 2020 with the CAP_2018–2020_ implementing more targeted NOx reduction measures^13,15,61^.

In order to investigate and quantify the CNY and CAP effects on the NO_2_ emission reduction in more detail, we now compare the evolution of the mean NO_2_ concentrations over 2015–2019 from both CAMSRA and observations. As shown in Fig. 1f, the observed NO_2_ concentrations in 2020 show a similar (or only slightly larger) decrease during the CNY as during the equivalent time period averaged over 2015–2019 but did not show a rapid recovery after day +6, revealing the emerging effect of the COVID-19 lockdown towards the end of the CNY. Therefore, the COVID-19 lockdown did not significantly modulate the NO_2_ reductions when compared to the CNY effect in earlier years, except from day +6 onward. We now can separate the CNY effect from the COVID-19 lockdown effect on NO_2_ decreases. From the daily variation of NO_2_ in 2019 (Fig. 1e), we may conclude that the most significant effect of the CAP has lasted 28 days after the CNY’s day when compared to CAMS’s expected emissions, and became weaker thereafter. Therefore, to accurately quantify the effect of COVID-19 lockdown measures on the NO_2_ deduction, the contribution of the CAP measures should also be excluded from the emission reduction during the CNY period and Extended COVID-lockdown period in 2020. Here, the effect of the CAP_2018–2020_ can be calculated by averaging the difference between CAMSRA and observed NO_2_ during the Before CNY period in 2018, 2019, and 2020 (Fig. 1f).

Equivalent to Fig. 1, daily variations of the CAMSRA O_3_ and the observed O_3_ in the time period 2015–2020 are displayed in Supplementary Fig. 1. It is found that the daily concentrations of the CAMSRA O_3_ went up and down following the evolution in the observed O_3_ in 2015–2020 extremely well. Nevertheless, there is a tendency of CAMSRA to overestimate O_3_ Before CNY and underestimate O_3_ during CNY and after CNY. This concomitant rise of O_3_ over the whole research period in each year might be interpreted by the unbalanced emission reduction strategy of ozone’s reaction precursors under the CAP_2018–2020_, most obvious in 2020, when the COVID*-*lockdown led to further NO_2_ reductions. Recent studies demonstrated that NOx emission reductions would lead to less O_3_ being consumed via NO titration, which could explain the increases of O_3_ during that period not only in China^62^ but also across Europe^63^.

### Quantifying the anthropogenic emission reduction at the city level

**Fig. 2:**
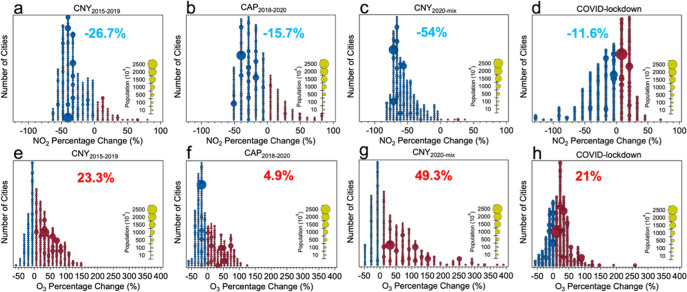
City number distributions of pollutant changes for anthropogenic drivers. The drivers include the CNY_2015–2019_ (Chinese New Year) effect for NO_2_ (**a**) and O_3_ (**e**), the CAP_2018–2020_ (Clean Air Plan) effect for NO_2_ (**b**) and O_3_ (**f**), the CNY_2020-mix_ effect for NO_2_ (**c**) and O_3_ (**g**), and the COVID-lockdown effect for NO_2_ (**d**) and O_3_ (**h**) in each city across China. Red solid circles represent cities with increased percentages, blue solid circles represent cities with decreased percentages. The size of the circles represents each city’s population.

Table 1 provides an overview of the different steps taken to disentangle and ultimately quantify the different anthropogenic and meteorological drivers, with the calculation described in more detail in “Methods”. Figure 2 shows the city number distributions as a function of the percentage changes in NO_2_ and O_3_ concentrations attributable to the different anthropogenic drivers for each city, with the size of the circles indicating each city’s population. Figure 2a, e shows the results for the CNY_2015–2019_ effect (see Table 1, Driver number 1). Overall, the average NO_2_ reduction across all the 367 (except for 87 outliers) cities is −26.7% (one-sigma range of −51.7 to −1.7%) and the average increase in O_3_ is 23.3% (one-sigma range of −18.6 to 65.2%). Among all the cities, 84.9% of them show decreased NO_2_ (with a −34.7 ± 15.7% reduction). On the contrary, the O_3_ concentration increased in more than half of the cities (by 44.8 ± 36.2%). Notably, the NO_2_ concentrations in cities with a high-density population (>5 million) were all reduced, and the average reduction ratio was −32.4 ± 17.3%. At the same time, all these densely populated cities show percentage increases in O_3_ with an average value of 48.5 ± 27.6%. Increasing O_3_ concentrations in cities with dense populations following the decline of its precursor of NO_2_ indicate a VOC-limited chemistry regime^62^, which together with an unbalanced control of the precursors of O_3_, cannot alleviate O_3_ pollution.

**Table 1:** Summary of the different steps taken to disentangle the various drivers.

Driver number	Driver	Method	Time period from CNY’s day (X)	Year (Y)	Average contribution
1	CNY_2020-mix_ CNY_2015–2019_	[Obs. (YX)-CAMS(YX)]/CAMS(YX)*100%	−7 to +10	2020 2015–2019	NO_2_(CNY_2020-mix_) = −54.0% O_3_(CNY_2020-mix_) = 49.3% NO_2_(CNY_2015–2019_) = −26.7% O_3_(CNY_2015–2019_) = 23.3%
2	CAP_2018–2020_	[Obs. (YX) – CAMS(YX)]/CAMS(YX) *100%	−21 to −8	2018–2020	NO_2_ = −15.7% O_3_ = 4.9%
3	CNY_2020_	CNY_2020-mix_ – CAP_2018–2020_	−7 to +10	2020	NO_2_ = −38.3% O_3_ = 44.3%
4	COVID-lockdown	CNY_2020-mix_ – CNY_2015–2019_ – CAP_2018–2020_	−7 to +10	2020	NO_2_ = −11.6% O_3_ = 21.0%
5	Extended COVID-lockdown	[Obs. (YX)-CAMS(YX)]/CAMS(YX)*100% – CAP_2018–2020_	+11 to +42	2020	NO_2_ = −34.7% O_3_ = 22.7%
6	Met effect	[ML_x_(Met-2020) - ML_x_(Met-2015–2019)]/ML_x_(Met-2015–2019)]*100%	−1 to +42	2020	NO_2_ = 7.8% O_3_ = −0.9%

Figure 2b, f shows the city count distribution of percentage changes in NO_2_ and O_3_ concentrations attributable to the CAP_2018–2020_ measures (Table 1, Driver number 2). In total, the average NO_2_ reduction across all cities was −15.7 ± 28.1% (one sigma, also hereafter), while O_3_ had an average increase of 4.9 ± 38.9%. During all the cities in China, there were 213 cities with a decreased NO_2_ concentration and 120 cities with a rise in O_3_ concentration under the effect of the CAP_2018–2020_. It was also found that the average percentage reduction in NO_2_ in cities with a high-density population (>5 million) was −29.2 ± 11.6% while the change in O_3_ was 17.6 ± 29.4%. Compared with the city count distribution of NO_2_ and O_3_ under the CNY_2015–2019_ impact, the city count distribution under the CAP_2018–2020_ effect reveals fewer cities with reduced NO_2_ concentrations and increased O_3_ concentrations, indicating that the short-term change of productive and economic activity during the CNY_2015–2019_ has a more significant influence on the reduction of NO_2_ and the O_3_ production than that under the long-term effect of CAP_2018–2020_.

Figure 2c, g illustrates the city number distributions of the percentage changes in NO_2_ and O_3_ concentrations attributable to the CNY_2020-mix_ effect (Table 1, Driver number 1). Generally, the average NO_2_ reduction across all cities was −54 ± 19.4% and O_3_ showed an average increase of 49.3 ± 85.2%. These numbers are largely consistent with previous studies (see “Introduction”) even though the considered time period is somewhat different. Compared to the changes during the CNY period in 2020, the average NO_2_ reduction changed from −54 to −11.6% and the O_3_ increases were smaller by 28% (decreasing from 49.3 to 21%) when excluding the effect of the CAP_2018–2020_ and the CNY_2015–2019_ (Fig. 2d, h). It is important to note that not excluding the effect of CAP_2018–2020_ and CNY_2015–2019_ may lead to an overestimation (and wrong attribution of the NO_2_ reductions and O_3_ increases) under the COVID-lockdown measures.

After quantifying the COVID-lockdown effect in the CNY period of 2020 in each city across China, we also explored the anthropogenic emission change during the Extended COVID-lockdown period. Supplementary Fig. 2a, c demonstrates the city count distribution of the percentage changes in NO_2_ and O_3_ concentrations attributable to the CAP_2018–2020_ and Extended COVID-lockdown effect, and Supplementary Fig. 2b, d shows the city distribution of the NO_2_ emission reduction and O_3_ change during the Extended COVID-lockdown period excluding the CAP_2018–2020_ effect (Table 1, Driver number 5). From Supplementary Fig. 2a, c, it was found that the average NO_2_ reduction across all cities is −50.4 ± 21.8%, and O_3_ has an average increase of 27.6 ± 43.1%. The emission reduction in NO_2_ is almost equal to the average value (52%) of the results from recent studies using ground-based observations across China. After excluding the effect of CAP_2018–2020_ from the Extended COVID effect (Supplementary Fig. 2a, c), the average NO_2_ reduction across all cities decreased significantly to −34.7 ± 16.9%, while the average O_3_ increase decreased to 22.7 ± 22.5%, which demonstrates that the isolated effect of the COVID-19 restrictions led to smaller than expected changes in NO_2_ and O_3_ concentrations.

### Meteorology-related emission changes

ML was finally used to quantify the potential influence of meteorological condition changes on the NO_2_ and O_3_ concentrations. Supplementary Fig. 3 shows the prediction of the NO_2_ daily concentration using the meteorological conditions in 2020 and the average meteorological condition in the equivalent period averaged over 2015–2019. It is found that the variations in the predicted NO_2_ using mean meteorological conditions from 2015 to 2019 are small, as expected for a climatological evaluation, in contrast to using the meteorological conditions of 2020, for which the results indicate a distinct impact of the meteorological factors on the variations in the NO_2_ concentrations. In particular, over much of the considered time period (Before CNY and up to day +19), prevailing meteorological conditions have led to above-average NO_2_ concentrations when compared to the climatology. Interestingly, starting at day 21 after the CNY day but lasting for a few days only, meteorology seems to lead to a short-lived decrease in the pollution situation.

**Fig. 3:**
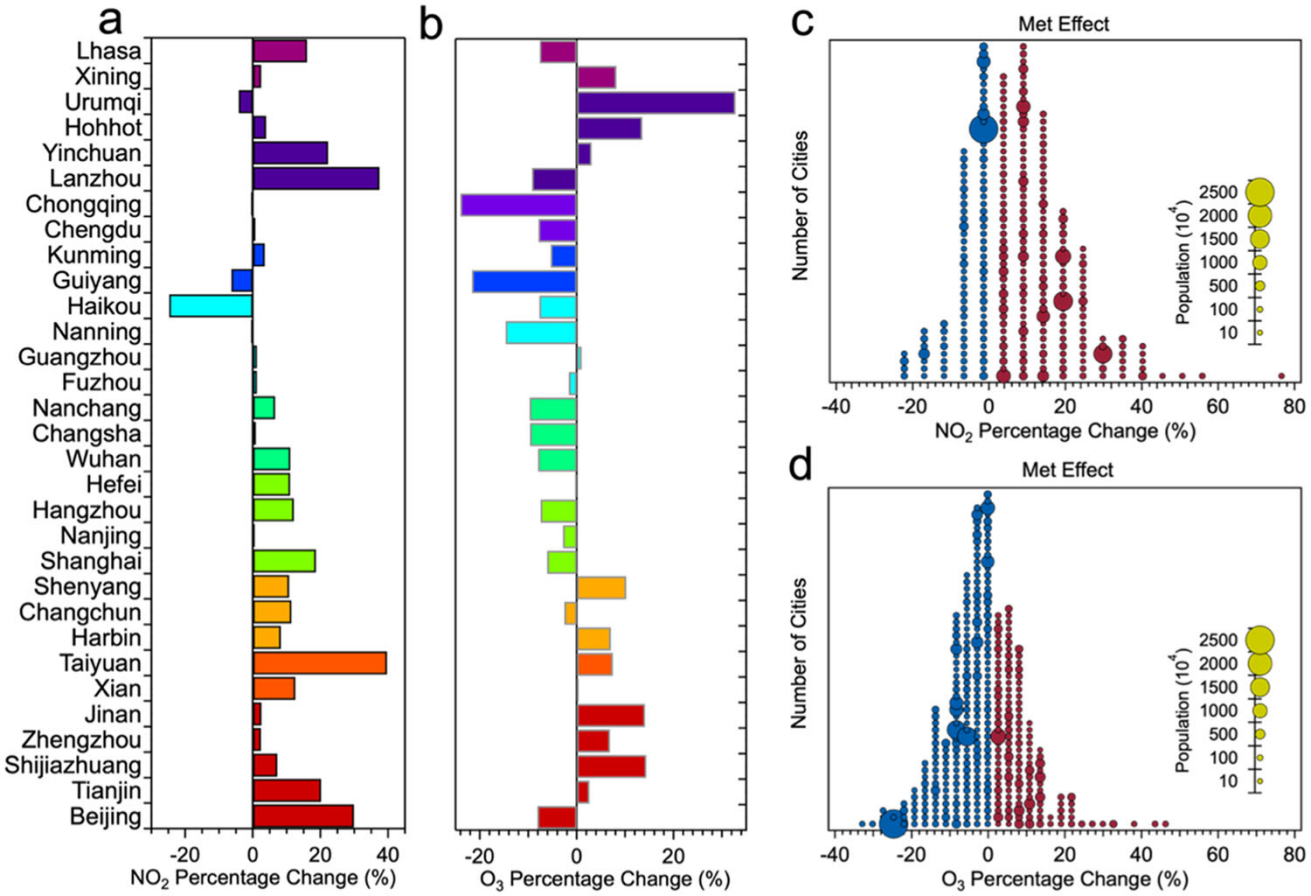
City-level NO_2_ and O_3_ changes attributable to meteorological drivers. **a**, **b** show the average NO_2_ and O_3_ changes in 31 capital cities in each province and **c**, **d** show the city number distributions of NO_2_ and O_3_ percentage changes attributable to the meteorological drivers in each city across China during the Total COVID-lockdown period, respectively. Red solid circles represent cities with increased percentages, blue solid circles represent cities with decreased percentages. The size of the circles represents each city’s population.

Figure 3 displays the average NO_2_ and O_3_ changes in 31 capital cities in each province (Fig. 3a, b) and the city number distribution of the percentage changes in the NO_2_ (Fig. 3c) and O_3_ concentrations (Fig. 3d) as attributed to the changes in meteorological conditions during the Total COVID-lockdown period. Overall, the average NO_2_ concentration increased by 7.8 ± 14% (but remains almost unchanged for O_3_ with a decrease of −0.9%), indicating meteorological conditions unfavorable to the transport and diffusion (clearing out) of NO_2_ during the Total COVID-lockdown period, although with only a small effect on O_3_. There are 87 of all cities and only 3 of 31 capital cities (Urumqi in Northwest China, Haikou in Pearl River Delta, and Guiyang in Yunnan-Guizhou Plateau), where the variations in the meteorological conditions are conducive to the transport and diffusion of NO_2_. Meanwhile, some studies also highlighted the influence of the specific surface meteorological conditions on the air pollution episodes in some capital cities^16^, including Beijing, Tianjin, Shijiazhuang, Jinan, Zhengzhou, Xi’an, Taiyuan, Shanghai, Guangzhou, especially Wuhan in Hubei Province, where about 50% of the pollution cases were related to atmospheric transport^17^.

### Health risk change during the COVID-19 lockdown

After quantifying the concentration changes of NO_2_ and O_3_ and attributing them to the different drivers of anthropogenic emissions and meteorological condition changes, we extend our methodology to also consider other air pollutants such as PM_2.5_, PM_10_, SO_2_, and CO in order to evaluate the health risk related to the overall air-quality change due to the COVID-19 lockdown restrictions (although without considering the increased health risk caused by the aerosol transmission of viruses during the pandemic^64,65^). The predicted average concentrations of the other pollutants in 367 cities and the model performance of each pollutant in 31 capital cities are shown in Supplementary Figs. 4–10, respectively. After predicting the concentrations of the six air pollutants during the COVID-19 lockdown, the resulting Excess Risks (ERs) in the two scenarios of the BAU and the COVID-19 lockdown can be calculated based on the World Health Organization (WHO) guidelines (released in 2021) and the Chinese Ambient Air Quality Standard grade II (CAAQS-II) standard (Supplementary Table 2), respectively.

Overall, when making a comparison of the ER differences (Supplementary Figs. 11–15) from the six air pollutants averaged over the 31 capital cities, these were higher for NO_2_ (−2.2%) than for PM_2.5_ (−0.77%), PM_10_ (−1.03%), SO_2_ (−0.05%), CO (0), and O_3_ (0.1%), indicating a significant ER decrease from NO_2_, PM_2.5_, PM_10_, and SO_2_ changes which also significantly offset the increased ER from O_3_ under the COVID-lockdown measures. These results are in stark contrast to those when ERs are being calculated based on the CAAQS-II standard. In this case, we find no ERs from SO_2_, NO_2_, O_3_, and CO because the concentrations of those four pollutants did not exceed the daily CAAQS-II standards. Rather, PM_2.5_ and PM_10_ (Supplementary Figs. 16 and 17) were the two main contributors to ERs during the COVID-19 lockdown.

**Fig. 4:**
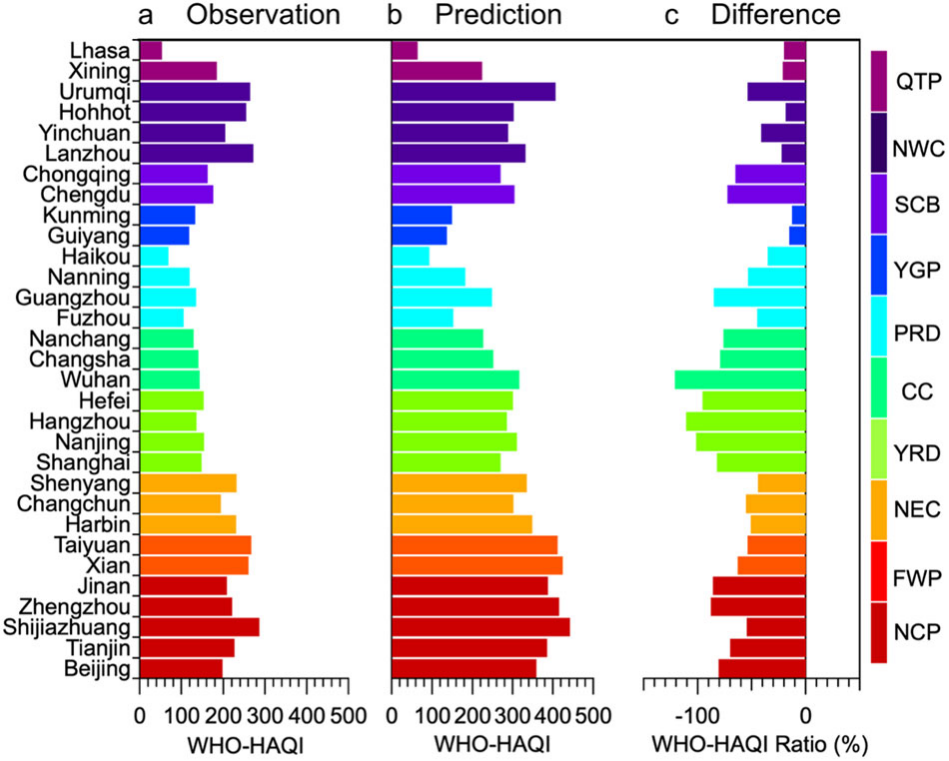
WHO-HAQI derived from observations and the counterfactual prediction. **a**–**c** represent the observed and predicted WHO-HAQIs, and the relative difference between them for 31 capital cities in each province across China. Different colors represent different regions in China, containing clusters of the 31 capital cities in each province according to their geographic locations.

To investigate the health benefits attributable to air-quality change, we also made a comparison of observed HAQI and predicted HAQI in 31 capital cities of China based on the WHO guidelines (hereafter as WHO-HAQI, Fig. 4) as well as the CAAQS-II standards (hereafter as CAAQS-HAQI, Supplementary Fig. 18). Overall, during the BAU period (Fig. 4b), the WHO-HAQI averaged over the 31 capital cities was 287, which is 1.8 times higher than the CAAQS-HAQI (102) (Supplementary Fig. 18b). After implementing the COVID-19 lockdown measures, the air quality improved significantly and the average WHO-HAQI (Fig. 4a) decreased to 179, with an average decline of 61% (Fig. 4c). For CAAQS-HAQI, the average HAQI decreased to 75 (Supplementary Fig. 18a), with an average decline of 21% (Supplementary Fig. 18c) therefore putting the HAQI into the category of “good” for the public’s health. Although the WHO-HAQI reduction is almost three times as high than that in the CAAQS-HAQI, the average WHO-HAQI after the reduction was still in the category “unhealthy” for the public’s health. Still a significant improvement in health benefits related to air quality could be attributed to the impact of COVID-19 restrictions coupled with that of the CAP and CNY.

To quantify how much the WHO-HAQI responded to these drivers regionally, the WHO-HAQI percentage changes between the COVID-19 lockdown period and BAU period in each region (see detailed information in Supplementary Table 3) were investigated. The results show that the WHO-HAQI percentage changes were negative in all the regions (Supplementary Fig. 19), including YRD (−98%), CC (−93%), NCP (−76%), SCB (−69%), FWP (−59%), PRD (−55%), NEC (−50%), NWC (−34%), QTP (−20%), and YGP (−14%). On the other hand, the CAAQS-HAQI percentage changes showed negative changes only in NCP (−38.57%), YRD (−35.74%), FWP (−33.45%), CC (−33.1%), SCB (−21.83%), NEC (−20%), PRD (−9%), and NWC (−4%), while they increased in YGP (0.2%) as well as for Haikou in PRD (10.66%). The differences in the results for the WHO-HAQI and CAAQS-HAQI can be explained once again by the fact that more air pollutants, that is SO_2_, NO_2_, and O_3_, are all contributing to the WHO-HAQI increases, whereas the calculation of the CAAQS-HAQI is only sensitive to PM concentration levels. Meanwhile, the increased concentrations of air pollutants can offset the health benefits from the reduction of other air pollutants in some regions/cities and for the two health standards (WHO and CAAQS) in different ways. For example in YGP, increased PM concentration transported from southwest Asia led to a positive difference in the CAAQS-HAQI^66^ (see also Supplementary Fig. 18), while these increases were compensated for by the reductions in NO_2_ leading to an overall negative difference in WHO-HAQI.

**Fig. 5:**
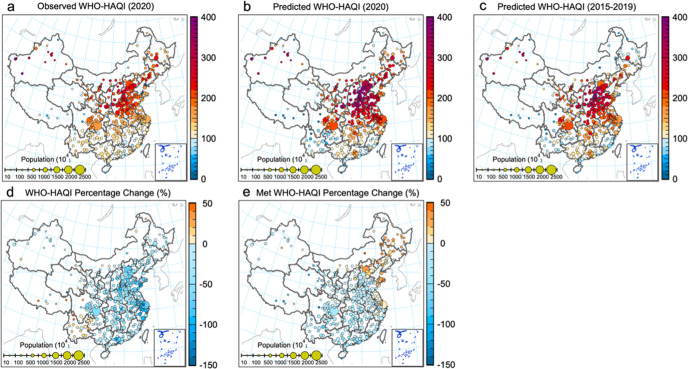
Spatial distributions of WHO-HAQI and attributed changes across China. **a**–**c** show the WHO-HAQI distributions as derived from observations in 2020, from a counterfactual prediction in 2020, and the predictions averaged over 2015–2019, respectively. **c**, **d** show the differences between observation and prediction in 2020, and the changes attributable to meteorology (Met), respectively. Solid circles in hot color represent cities with increased percentages, and in cold color cities with decreased percentages. The size of the circles represents each city’s population.

Furthermore, the spatial distribution in WHO-HAQI differences between the observations (Fig. 5a) and the prediction (Fig. 5b) in all cities was also investigated (Fig. 5d). It was found that WHO-HAQIs in most of the cities (92.9%) in China were reduced by an average relative amount of 55.43 ± 26.97% under the combined impacts of CAP, CNY, COVID-19 restrictions, and meteorological drivers, with those cities having successively announced travel bans within days, including restrictions on non-essential activities, suspension of travel between cities, and closure of all factories. Cities with increased WHO-HAQIs (7.1% of all cities) (Fig. 5d) are mainly located in YGP and inland in the northwest (like parts of Inner Mongolia, Gansu Province, and QTP Region). These areas with increased PM concentrations were frequently affected by polluted air flows from other regions and also local sources (like sandstorms or enhanced indoor coal heating) during the COVID*-*lockdown, respectively^28^.

At last, to estimate the meteorological effect (Met effect) on the variation of the WHO-HAQI, the spatial distribution of WHO-HAQI differences attributable to the meteorological change between that in 2020 and averaged over 2015 to 2019 was investigated (Fig. 5e). WHO-HAQIs in 188 cities (51.5% of all cities) experienced an increase under the meteorological conditions in 2020 (Fig. 5b) when compared to 2015–2019 average conditions (Fig. 5c). Increased WHO-HAQIs (Fig. 5e) in those cities were mainly located in the NEC, Inner Mongolia, and part of NCP, YRD, indicating that the meteorological conditions in 2020 have deteriorated air quality in those regions. However, there were still some cities in NCP and YRD with decreased WHO-HAQIs, indicating that unfavorable meteorological conditions in 2020 cannot offset the health benefits of emission reductions in those regions. On the other hand, somewhat less than half of all the cities in CC, PRD, SCB, YGP, and TQP benefitted from decreased WHO-HAQIs, demonstrating an improved air quality in those regions under the meteorological conditions in 2020.

Overall, the nationwide lockdown measures taken to reduce the spread of COVID-19 had an impact on air pollutant concentrations across China. However, the actual response of air pollutant concentrations and related health risks to the COVID-19 restrictions cannot be quantified in a straightforward way because multiple drivers, such as meteorological conditions, policy regulations such as the CAP, and the CNY celebrations, also have an effect on air quality that confounds the attribution of the changes to COVID-19^61,67,68^.

This study aimed at disentangling the different drivers of observed air pollutant changes during the COVID-19 lockdown period in 2020. In particular, it quantified the changes in NO_2_ and O_3_ at the city level across China attributable to the effect of the yearly occurrence of CNY, which coincided with the lockdown measures, and also characterized the contributions of the CAP and the COVID-19 lockdown restrictions to the NO_2_ concentration change separately, based on a combined model-measurement approach using the CASMRA dataset and surface observations from 2015 to 2020. It finally evaluated the impact of the 2020 meteorological conditions on the variations of NO_2_ and O_3_ concentrations during the outbreak of COVID-19 using a ML method, and at last explored the health risk change relating to the varying air quality under the COVID-19 lockdown. Our results indicate that both the CNY effect and the new CAP had a considerable influence on the NO_2_ emissions from 2015 to 2019. Compared to the CNY in the equivalent time period during 2015–2019, the CNY effect in 2020 lasts longer, which is attributed to the COVID-19 restrictions. The average anthropogenic emissions of NO_2_ across China under the impact of the CNY and the CAP decreased by −26.7% and −15.7%, respectively. The latter has not been previously determined but is important to highlight since it reflects the effectiveness of China’s new CAP regulations. Excluding the effect of the CAP and CNY (and thus fully attributable to the COVID-19 lockdown), showed that the anthropogenic emissions of NO_2_ across China during the CNY in 2020 were reduced on average by an additional 11.6%. For the average anthropogenic emissions of NO_2_ across China during the Extended COVID-lockdown period, the reduction was 34.7% after excluding the effect of the CAP_2018–2020_. On the contrary, the average O_3_ concentration showed increases of 23.3% and 4.9% under the effect of the CNY and the CAP, and an increase of 21% and 22.7% attributed to the effect of COVID-lockdown and Extended COVID-lockdown, respectively. These estimated contributions to the total air pollutant change however neglect the impact of the meteorological condition changes in 2020, which also affect the NO_2_ variations. Our results reveal that the meteorological conditions prevailing in 2020 lead to an adverse effect and contribute to an increase in NO_2_ concentrations with an average value of 7.8% when compared to climatological 2015–2019 conditions. At last, we also evaluated the health risk related to the air-quality change during the Total COVID period. Our results demonstrated that the reduction of ER from NO_2_ was the main driver of the derived health benefit and was able to offset the ER increase from O_3_. Overall, WHO-HAQIs were reduced on average by −51.4% in all cities across China. However, changes in meteorological conditions deteriorated the WHO-HAQI in NEC significantly, and local governments will need to adopt to account for such changes using more scientific emission reduction measures to reduce health risks in these regions, in particular in the light of a changing climate which is expected to lead to aggravated changes in meteorological variables.

## Methods

### Station data of air pollutants

The station data of air pollutants including PM_2.5_, PM_10_, SO_2_, NO_2_, O_3_, and CO in 367 cities (except those in Taiwan, Hong Kong, and Macau, no data in Laiwu from 2019 to 2020) (see Supplementary Fig. 20) were released by China’s National Environmental Monitoring Center (NEMC) (http://www.cnemc.cn). Daily concentrations of the six air pollutants in each city were calculated by averaging the hourly data from January 1, 2015, to April 30, 2020. Data quality control was executed for different measurement stations as in the previous study^2^.

### Station data of meteorological parameters

The hourly surface meteorological observation data including temperature (T, °C), relative humidity (RH, %), wind speed (WS, m/s), wind direction (WD, °), precipitation (Pre, mm), pressure (P, hPa) from 2015 to 2020 across China are used as input variables to the ML model. There are a total of 2425 National Meteorological Stations nationwide (excluding Zhongshan Station and Great Wall Station in Antarctica) with their geographical locations shown in Supplementary Fig. 21. However, we only selected the 367 stations with locations closest to the air pollutant monitoring sites available. Meteorological data can be downloaded from the National Meteorological Science Data Center (https://data.cma.cn/).

### CAMSRA dataset

NO_2_ and O_3_, obtained from the Copernicus Atmosphere Monitoring Service Reanalysis (CAMSRA) and produced by the European Centre for Medium-Range Weather Forecasts (ECMWF), are used for comparison with the surface observation data. CAMSRA used an emission inventory that did not represent the COVID-19 lockdown and CNY emission decreases, nor the reductions made under the two CAPs. While CAMSRA assimilates satellite retrievals of tropospheric NO_2_ and O_3_^69^ and therefore in principle should be corrected toward the “real world”, the assimilation is not able to correct the surface concentrations of the model field, mainly due to the large impact of the emissions (which were not updated as mentioned below) and the limited information content of the assimilated satellite retrievals (due to broad averaging kernels, spatial, and temporal coverage). Thus, this simulation can be used as a counterfactual for a world in which the lockdown or air-quality regulations did not happen. For CAMSRA NO_2_ and O_3_, the temporary resolution is 3 hours and the spatial resolution is 0.75 × 0.75 at 60 vertical model levels. Anthropogenic emissions used to drive CAMSRA were based on a modified MACCity inventory, and monthly mean VOC emissions were calculated by the MEGAN model using MERRA reanalyzed meteorology for 2003–2016^69^. The CAMSRA dataset can be downloaded from the Atmosphere Data Store (https://ads.atmosphere.copernicus.eu/cdsapp#!/home) and has been used to disentangle contributing factors for NO_2_ changes in Europe in spring 2020^70^.

Before evaluating the anthropogenic emission reduction, a comparison between CAMSRA and the observational dataset was conducted to test whether CAMSRA can capture observed NO_2_ variations. To this end, the gridded CAMSRA NO_2_ is first interpolated in longitude and latitude onto each measurement station in the 367 cities in China to get time-series concentrations of CAMSRA NO_2_ from 2015 to 2019 at these locations. The time series of CAMSRA NO_2_ and observed NO_2_ in each city were then fitted by using the linear fitting method to calculate the slope (S) and the Pearson Correlation Coefficient (PCC). A filter window (0.5 < S < 1.5 and PCC > 0.2) was applied to filter out outliers. The same procedure was then applied to evaluate CAMSRA ozone with help of the observational ozone. The focus on NO_2_ and ozone is here justified by the findings of a range of studies that O_3_ pollution has become more serious in China as a result of unbalanced air pollutant control measures, which focused on the reduction of PM_2.5_ and NOx. Using the same filter window as for NO_2_, there are a total of 87 cities that can be treated as outliers. After removing these outliers, the CAMSRA NO_2_ and O_3_ match the NO_2_ and O_3_ observations well, with average PCCs of 0.51 and 0.64 for NO_2_ and O_3_, respectively (Supplementary Fig. 22).

### Machine-learning model

We used a ML model rather than a chemical transport model, because the latter’s performance can be limited by its spatial resolution and potentially outdated emission inventories^71^. The ML, on the other hand, is expected to capture such location-specific characteristics and thus is more suitable for the prediction of pollutants in the different cities across China, especially those located in the desert and plateau areas in the northwest of China or those lacking emission inventories. The dataset of ML model is always split into two parts: a training dataset and a test dataset. In this paper, meteorological and time variables from 2015 to 2019 are selected as the training dataset of the ML model to predict the concentration of six air pollutants in the first 3 months of 2020. The time variables are listed as follows: Julian day, day of the week, hour of the day, the CNY days in each year, and the date index.

Due to the complex nonlinear relationship between weather conditions and air pollutants, this study used the Gradient Boosting Machine (GBM), which is the latest ensemble method based on a decision tree, to predict the concentration of air pollutants in 2020. There are several outstanding advantages of using GBM^71^. First, GBM can implement the feature selection internally, which ensures the model avoids a strong drop in the prediction skill when selecting potentially useless features. Furthermore, information on the importance of different features can be provided by GBM. At last, compared with general parametric methods, the GBM, a nonparametric method based on decision trees, generally operates depending on splitting a mother tree into two different branches, which is beneficial to design one model with high work efficiency.

To select the best ML model, the time-series split rolling method is selected to execute the cross-validation before the implementation of the ML prediction. Since the features, like temperature, and pressure used in this study, are temporal variables. These cannot be considered as independent data points due to the occurrence of autocorrelation. To account for this autocorrelation, we execute the so-called time-series cross-validation with four experiments, a method also applied in a similar study^72^ with a focus on Europe/Spain. The time-series split rolling cross-validation was with five splits, in which data used for training always precedes the data used for validation. In detail, training ML models are over 2015, 2015–2016, 2015–2017, 2015–2018, 2015–2019, and testing them over the 3 first months of 2016, 2017, 2018, 2019, and 2020, respectively. The cross-validation results are shown in Supplementary Fig. 23 and the details of the performance scores for each experiment are listed in Supplementary Table 4. After cross-validation, the root-mean-squared error (RMSE) and PCC are calculated to evaluate the ML model’s performance. Generally, the highest performance of a model is found at a minimum RMSE and a maximum PCC, with the values of the latter approaching 1. The performance scores (Supplementary Fig. 24) for the training dataset are: RMSE = 6.9 μg m^−3^, PCC = 0.85; and the prediction performance scores are: RMSE = 13.2 μg m^−^^3^, PCC = 0.71, which are close to the prediction performance of the study in Spain^72^.

### Methodology

In the following sections of evaluation of the combined effect, the CNY effect, and the CAP effect, the logical reasoning behind the approach taken to derive different anthropogenic drivers of the observed NO_2_ decline during the first quarter of 2020 is given, while the following Section of evaluation of the meteorology effect provides an overview of how the meteorological driver is estimated. The section on the estimation of health effects explains the method to calculate the HAQI.

### Combined effect of anthropogenic drivers

As mentioned above, the advantage of the CAMSRA simulation used in this study is that it reflects a counterfactual to the real world that does not include emission reductions due to the CNY, CAP, or COVID-lockdown. The difference in air pollutant concentrations between CAMSRA and the observations (see Fig. 1a–f) can thus be attributed to the total influence of changes in anthropogenic activities. This overall difference can, in the next step, be attributed to single drivers, the CNY, CAP, and COVID-lockdown effects as explained in the following.

### Calculation of the CNY and CAP effect

The *CNY* day is defined according to the lunar calendar and varies from a date in late January to early February over the time period 2015–2020. Usually, the CNY effect begins approximately one week before the CNY’s day and then lasts for ~10 days after the CNY’s day (as derived from the years 2015–2019) and this time period is hereafter labeled as “*CNY*“. The 2 weeks before the CNY (days −21 to −8 from the CNY’s day) is here defined as the “Before CNY” period. One month post the CNY (days +11 to +42 from the CNY’s day) is called the “Extended COVID-lockdown” period. And the Total COVID-lockdown period can be defined from 1 day before the *C*NY’s day to one month post the CNY (or days −1 to +42 from the CNY’s day). The different periods in 2020 are represented by different background colors in Fig. 1, Supplementary Figs. 1 and 3.

To this end, we first calculate the difference between the average NO_2_ simulated by CAMS and observed in 2015–2019 (Fig. 1f), which was defined as the CNY_2015–2019_ effect and calculated from the equation in Table 1, Driver number 1). It should be noted that Table 1 has listed all the target contributors, the methodological approach, and the time period considered for these target contributors that appeared in this paper. In a second step, the COVID-lockdown effect (Table 1, Driver number 4) can be estimated to a first approximation as the NO_2_ difference between the observations in 2020, minus the average CNY_2015–2019_ effect as calculated above. However, as investigated in Fig. 1d–f, this interpretation would lead to an overestimate of the COVID-lockdown effect since it neglects the impact of the CAP_2018–2020_ on NO_2_ concentrations that have not yet been accounted for in the CAMS emissions (nor in previous studies). Thus, to estimate the real COVID-lockdown effect accurately, the CAP_2018–2020_ effect should also be excluded from the second step approximation.

### Evaluation of the meteorology effect (Met effect)

After evaluating the anthropogenic emission changes under the isolated effects of the CNY, the CAP, and the COVID restrictions, ML was used to estimate the Met effect on the NO_2_ and O_3_ concentration change. To quantify the variations of NO_2_ and O_3_ concentration under the Met effect, two ML experiments were executed. The first (baseline) used meteorological and time variables from 2015 to 2019 as the training dataset of the ML model to predict the concentrations of NO_2_ and O_3_ in the first three months of 2020, and the second applied this predictive model based on the independent features during the equivalent time period averaged over 2015–2019 to predict NO_2_ and O_3_ concentrations in 2020. The difference between the predicted NO_2_ (or O_3_) derived from the independent features in 2020 and for the equivalent time period averaged over 2015–2019, and based on the same predictive model, can represent the Met effect on the variation of NO_2_ (O_3_) (Table 1, Driver number 6).

To quantify the variation of health risks from six air pollutants under the impact of the Met effect in the next section, we also executed the same two experiments by using the ML model for PM_2.5_, PM_10_, SO_2_, and CO respectively.

### Estimation of health effects

COVID-19 lockdown measures can lead to a change in air quality. As a response, the health effect of all six air pollutants also varies under those restriction measures. In this paper, the excess risk (ER) from each pollutant is evaluated as well as the Health-based Air Quality Index (HAQI). The relative risk (RR) function of air pollutants is generally expressed by an exponential linear function (Eq. (1)). The HAQI is an index that sets a threshold concentration of pollutants. It assumes that there is no health risk for air pollutants below the threshold concentration. Therefore, only when the concentration of pollutant exceeds a given threshold concentration will there be an ER of death (Eq. (2)). In general, considering that the calculation of ER is directly related to the threshold concentration C_0_, we use the WHO guideline/CAAQS-II as the upper limit for six air pollutants to evaluate the ERs and the HAQI in each city across China.

1 $$RR_i = \exp \left[ {\beta _i\left( {C_i - C_{i,0}} \right)} \right],C_i \,>\, C_{i,0}$$

2 $$ER_i = RR_i - 1$$

3 $$ER_{total} = \mathop {\sum }\limits_{i = 1}^n ER_i = \mathop {\sum }\limits_{i = 1}^n \left( {RR_i - 1} \right)$$

4 $$RR^ \ast = ER_{total} + 1 = \exp \left[ {\beta \left( {C^ \ast - C_0} \right)} \right]$$

5 $$C_i^ \ast = \ln \left( {RR^ \ast } \right)/\beta _i + C_{0,i}$$

In Eq. (1), $RR_i$ represents the relative risk of pollutant *i*, $\beta _i$ represents the exposure-response coefficient of pollutant *i*, which means the additional risk of death caused by air pollutant increased by each unit concentration; $C_{i,0}$ is the threshold concentration of pollutant *i*. According to an overview of the short-term exposure to air pollutants and daily mortality in China, the *β* value is 0.038%, 0.032%, 0.081%, 0.13%, and 0.048% when concentrations of PM_2.5_, PM_10_, SO_2_, NO_2_, O_3_ have additional unit concentration value (µg/m^3^). For CO, the *β* value is 3.7% per 1 mg/m^3^ increase. When ERs of six air pollutants are added to $ER_{total}$ as shown in Eq. (3), the equivalent concentration of $C_i^ \ast$can be calculated according to Eq. (5). Thus, HAQI can be derived similarly to AQI^73^ (calculation details can be found in Supplementary Note 1). Several studies^14,73,74^ have demonstrated that HAQI is more appropriate to estimate the health effect of multi-air pollutants. In particular, the HAQI takes into account the opposing effects on human health of NO_2_ decreases and ozone increases observed in many cities as a consequence of the COVID-lockdown. To calculate the HAQI, 8-h peak O_3_ was selected in the 8-h moving averaged concentration each day.

## Supplementary information

SUPPLEMENTAL MATERIAL

## Data Availability

The station data of air pollutants including PM_2.5_, PM_10_, SO_2_, NO_2_, O_3_, and CO in 367 cities in China are freely available at the China’s National Environmental Monitoring Center (http://www.cnemc.cn), and the nationwide meteorological station data can be downloaded from the National Meteorological Science Data Center (https://data.cma.cn/). The CAMSRA model data for the Machine Learning experiment used in this study is freely available at the Atmosphere Data Store (https://ads.atmosphere.copernicus.eu/cdsapp#!/home).

## Code availability

The python code and data used to run the GBM can be obtained upon request from the corresponding authors.
