# Supplementary material for: Disentangling drivers of air pollutant and health risk changes during the COVID-19 lockdown in China
Source: NPJ Clim Atmos Sci. 2022 Jun 30;5(1):54. doi: 10.1038/s41612-022-00276-0 (PMC9244310; doi:10.1038/s41612-022-00276-0)
Supplement: Supplementary file 1 — SUPPLEMENTAL MATERIAL [file 41612_2022_276_MOESM1_ESM.pdf]

Supplementary Materials

Disentangling Drivers of Air Pollutant and Health Risk Changes during the COVID-19 Lockdown in China

Fuzhen Shen[1,2 †], Michaela I. Hegglin[2,3* †], Yuanfei Luo[4 †], Yue Yuan[5], Bing Wang[6], Johannes Flemming[7], Junfeng Wang[1,8], Yunjiang Zhang[1], Mindong Chen[1], Qiang Yang[9], Xinlei Ge[1*]

[1]Jiangsu Key Laboratory of Atmospheric Environment Monitoring and Pollution Control, Collaborative Innovation Center of Atmospheric Environment and Equipment Technology, School of Environmental Science and Engineering, Nanjing University of Information Science and Technology, Nanjing 210044, China

[2]Department of Meteorology, University of Reading, Reading, RG6 6BX, UK

[3]Institute of Energy and Climate Research, IEK-7: Stratosphere, Forschungszentrum Jülich, 52425 Jülich, Germany

[4]Paradigm Inc., Beijing 100000, China

[5]Jining Meteorological Bureau, Shandong 272000, China

[6]Henley Business School, University of Reading, Reading RG6 6UD, UK

[7]ECMWF, Shinfield Park, Reading, RG2 9AX, UK

[8]John A. Paulson School of Engineering and Applied Sciences, Harvard University, Cambridge, MA 02138, USA

[9]Hongkong University of Science and Technology, Hong Kong 999007, China

*Corresponding author, Email: m.i.hegglin@reading.ac.uk； Email: caxinra@163.com

†These authors contributed equally to this work.

This file includes:

Supplementary Note 1
Supplementary Figures 1 to 24
Supplementary Tables 1 to 4

Supplementary Note 1

The AQI for each pollutant (AQI_i) was first calculated by using Eq. (1), and the maximum AQI_i of all pollutants was chosen as the overall AQI according to Eq. (2).

$$\boldsymbol{AQI_i = \frac{AQI_{i,j} - AQI_{i,j-1}}{(m_{i,j} - m_{i,j-1})} \times (m_i - m_{i,j-1}) + AQI_{i,j-1},\ j > 1},$$

$$\boldsymbol{AQI_i = AQI_{i,1} \frac{m_i}{m_{i,1}},\ j = 1} \qquad (1)$$

$$\boldsymbol{AQI = max(AQI_1, AQI_2 \cdots, AQI_n), n = 1, 2, \cdots, 6}, \qquad (2)$$

where i represents the pollutant i; m_i is the measured concentration of i; j is the health category index; $m_{i,j}$ is the reference concentration for pollution i corresponding to the jth health category. Within this system, the air quality is classified into six classes according to the ranges of AQI values (below 50: excellent, satisfactory; 51-100: good, acceptable; 101-150: light pollution, unhealthy for sensitive people; 151-200: moderate pollution, unhealthy; 201-300: serious pollution, very unhealthy - healthy people commonly have symptoms; beyond 300: very severe pollution, hazardous - healthy people have significant symptoms, and should avoid outdoor activities).

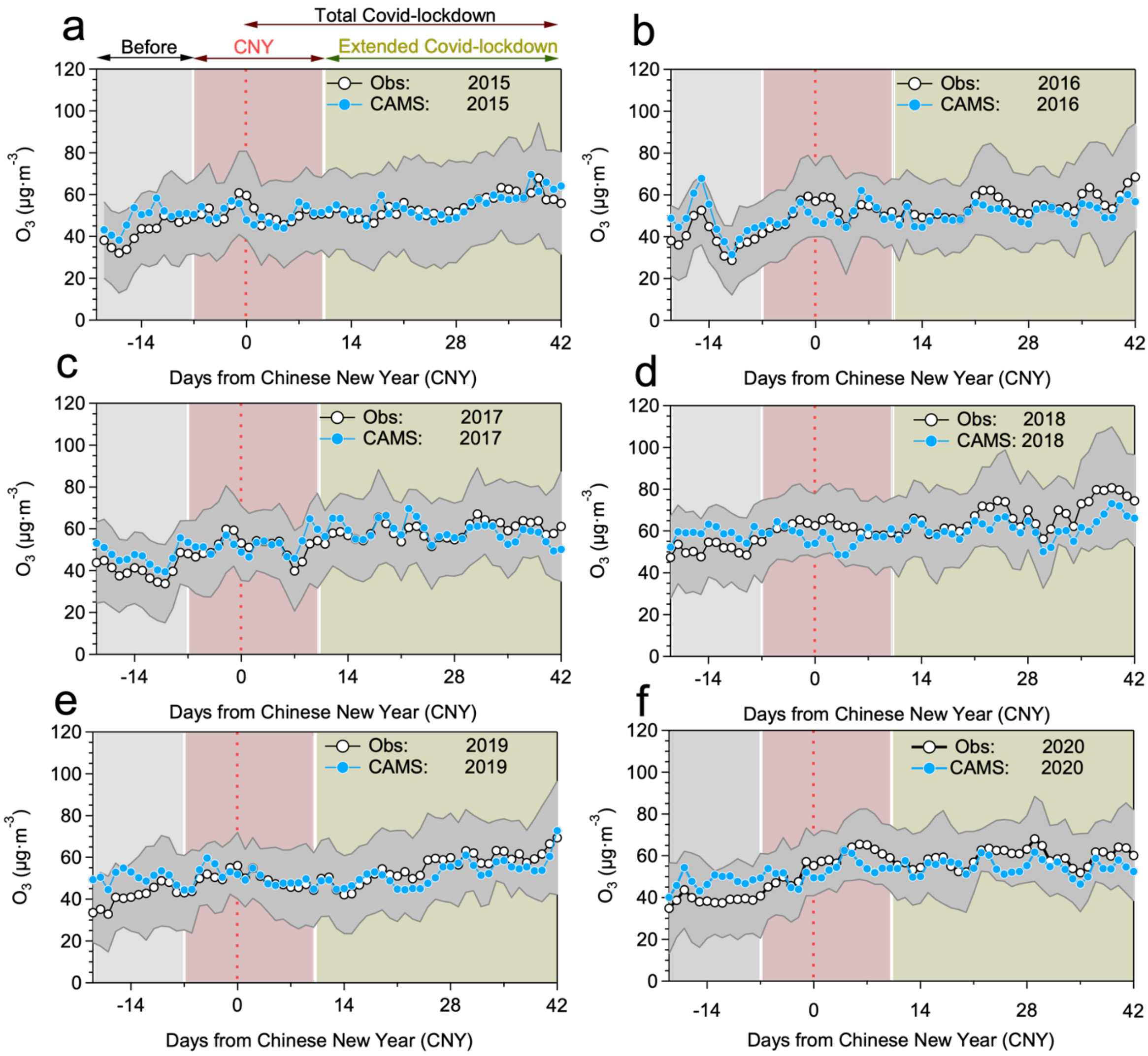

Supplementary Figure 1. Comparison between observations and CAMSRA for O_3 across China. The blue solid circle represents average daily O_3 concentration of CAMSRA, and the black circle represents average daily O_3 concentration of observation across China, respectively. The red dash line represents the Chinese New Year day. Different background colors represent different time period including 'Before CNY'(gray) ranged from days -21 to -8 from the CNY's day, 'CNY'(red) ranged from days -7 to +10 from the CNY's day, and 'Extended COVID-lockdown'(moss) ranged from days +11 to +42 from the CNY's day. The Total COVID-lockdown period ranges from days -1 to +42 from the CNY's day.

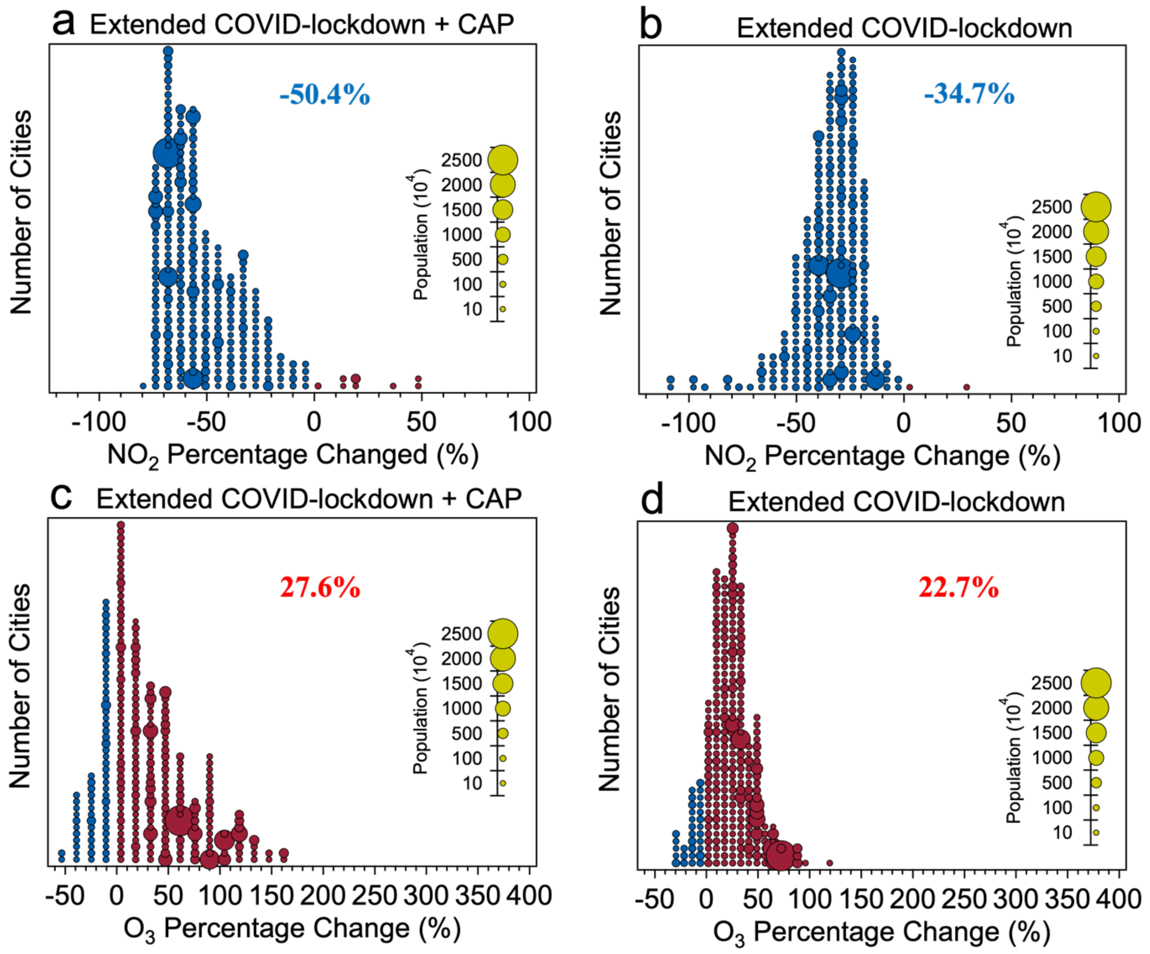

Supplementary Figure 2. City distributions of pollutant changes for anthropogenic drivers. Red solid circles represent cities with increased percentages, blue solid circles represent cities with decreased percentages. The size of the circles represents each city's population.

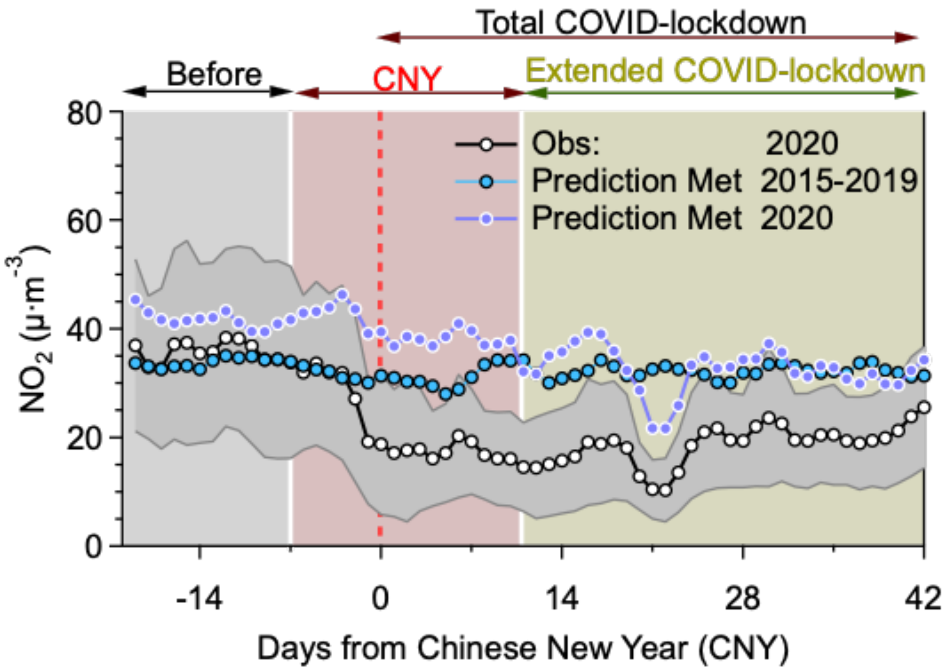

Supplementary Figure 3. Daily concentrations of averaged NO_2 in 367 cities across China. The black circle represents observed average daily NO_2 concentration across China; the blue solid circle represents prediction of average daily NO_2 concentration by using the meteorological conditions in 2020; the purple solid circle represents prediction of average daily NO_2 concentration by using the average meteorological condition in the equivalent period averaged over 2015 to 2019. The gray, red, moss colors represent different time period of Before CNY period, CNY period, and Extended COVID-lockdown period, respectively.

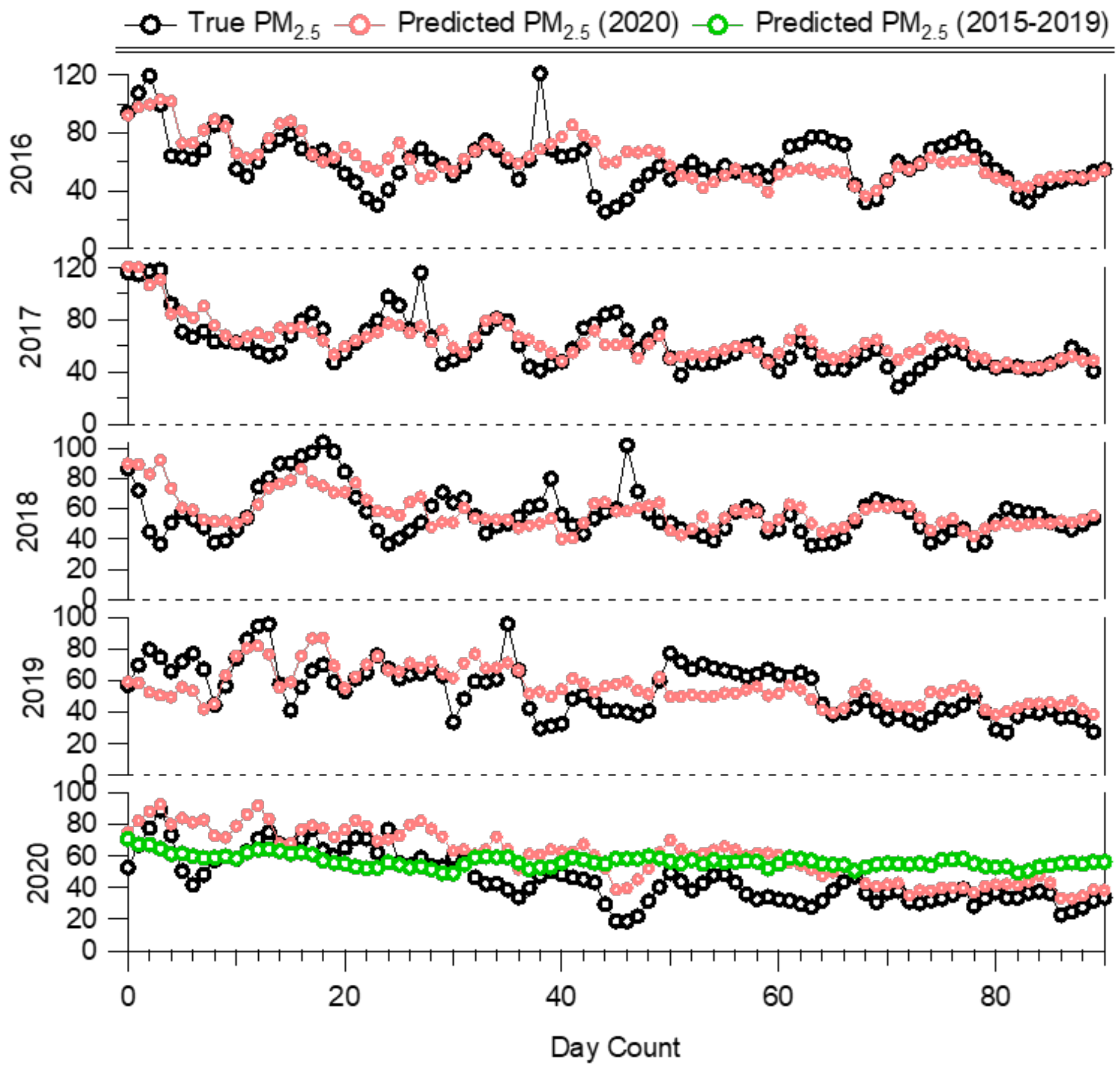

Supplementary Figure 4. Daily concentrations of $PM_{2.5}$ across China from 2016 to 2020. The black circle represents observed average daily $PM_{2.5}$ concentration across China; the red circle represents prediction of average daily $PM_{2.5}$ concentration using the meteorological conditions in 2020; the green circle represents prediction of average daily $PM_{2.5}$ concentration using the average meteorological condition in the equivalent period averaged over 2015 to 2019.

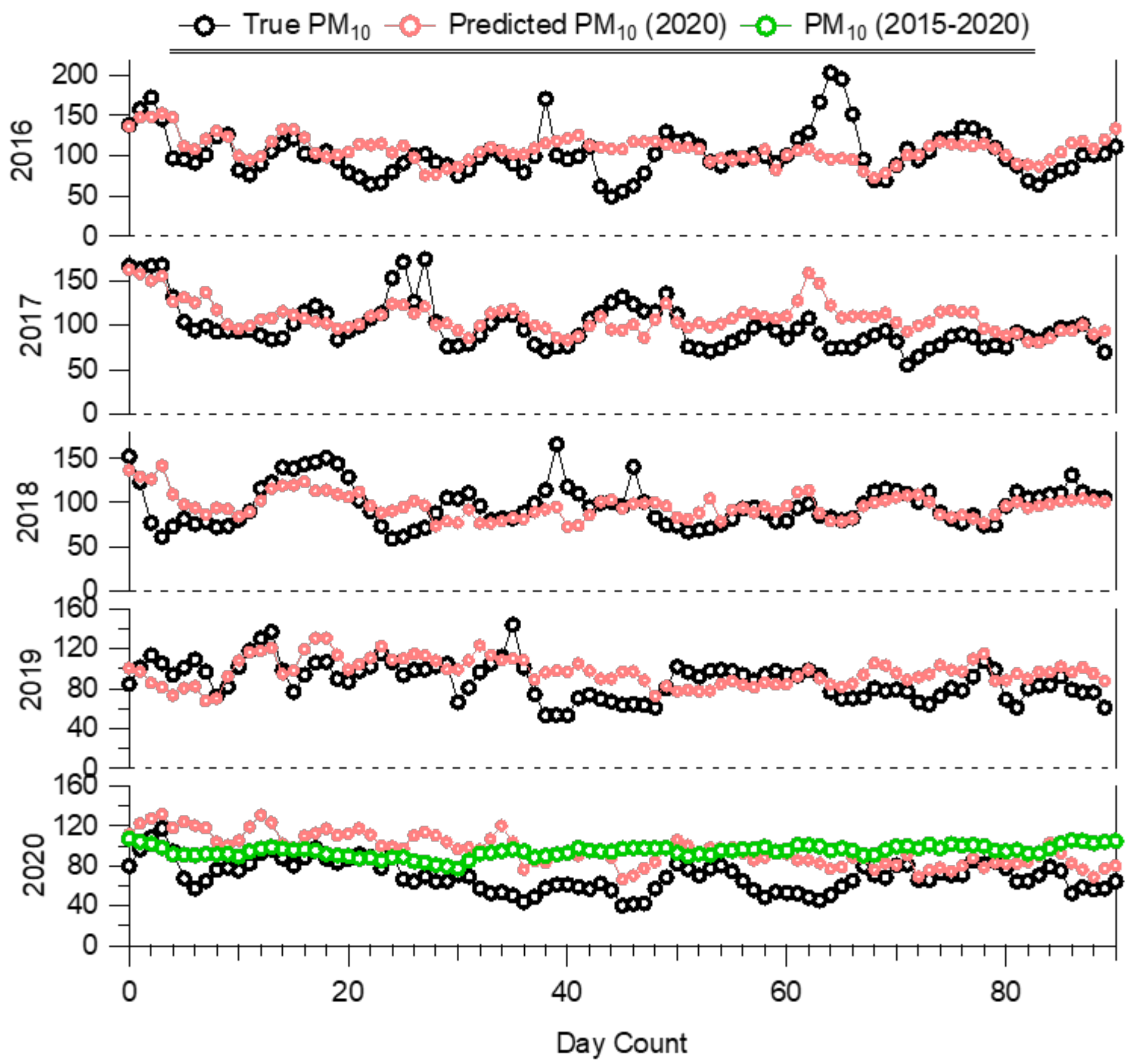

Supplementary Figure 5. Same as in Supplementary Figure 4, but for PM_{10}.

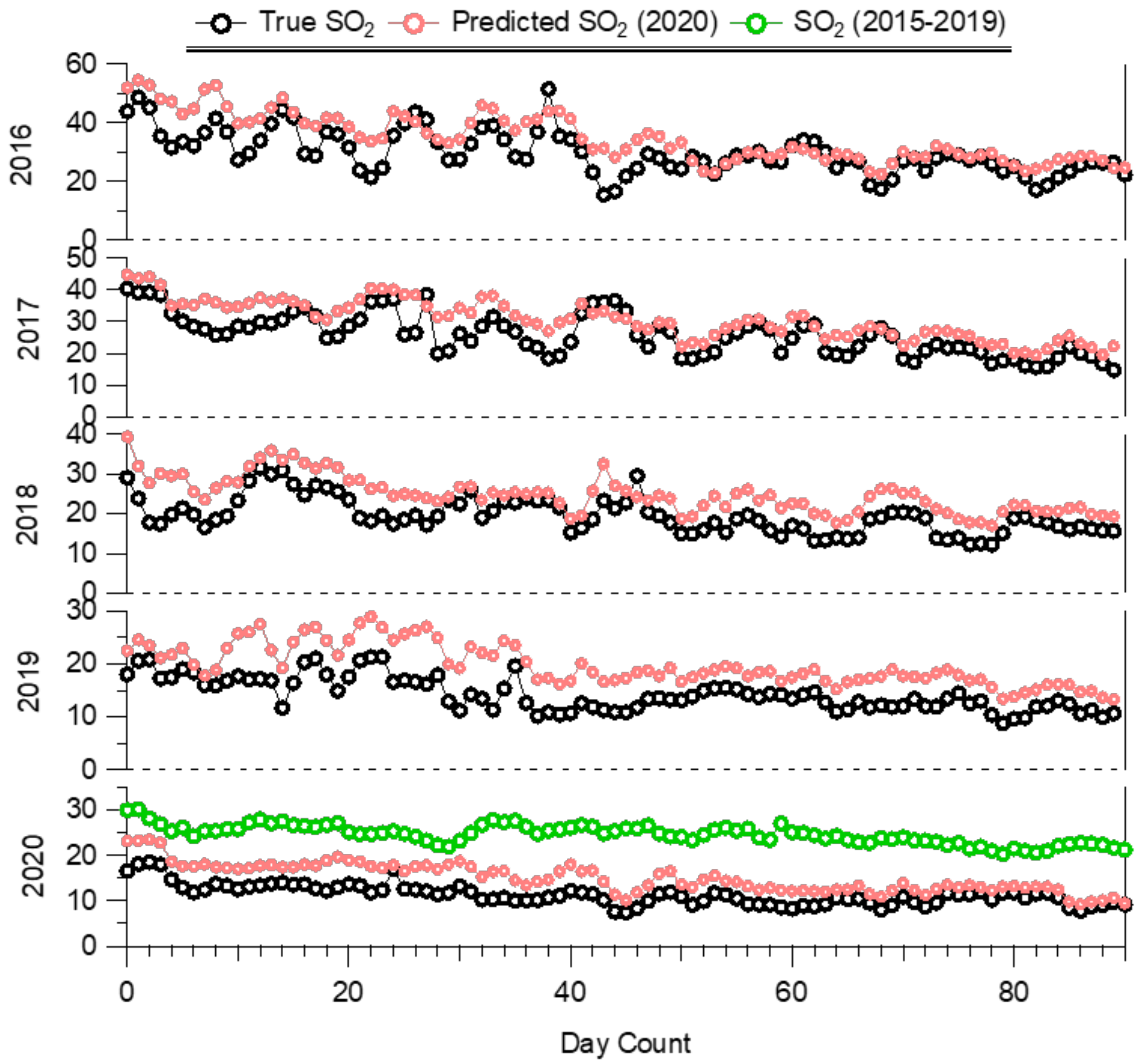

Supplementary Figure 6. Same as in Supplementary Figure 4, but for SO_2.

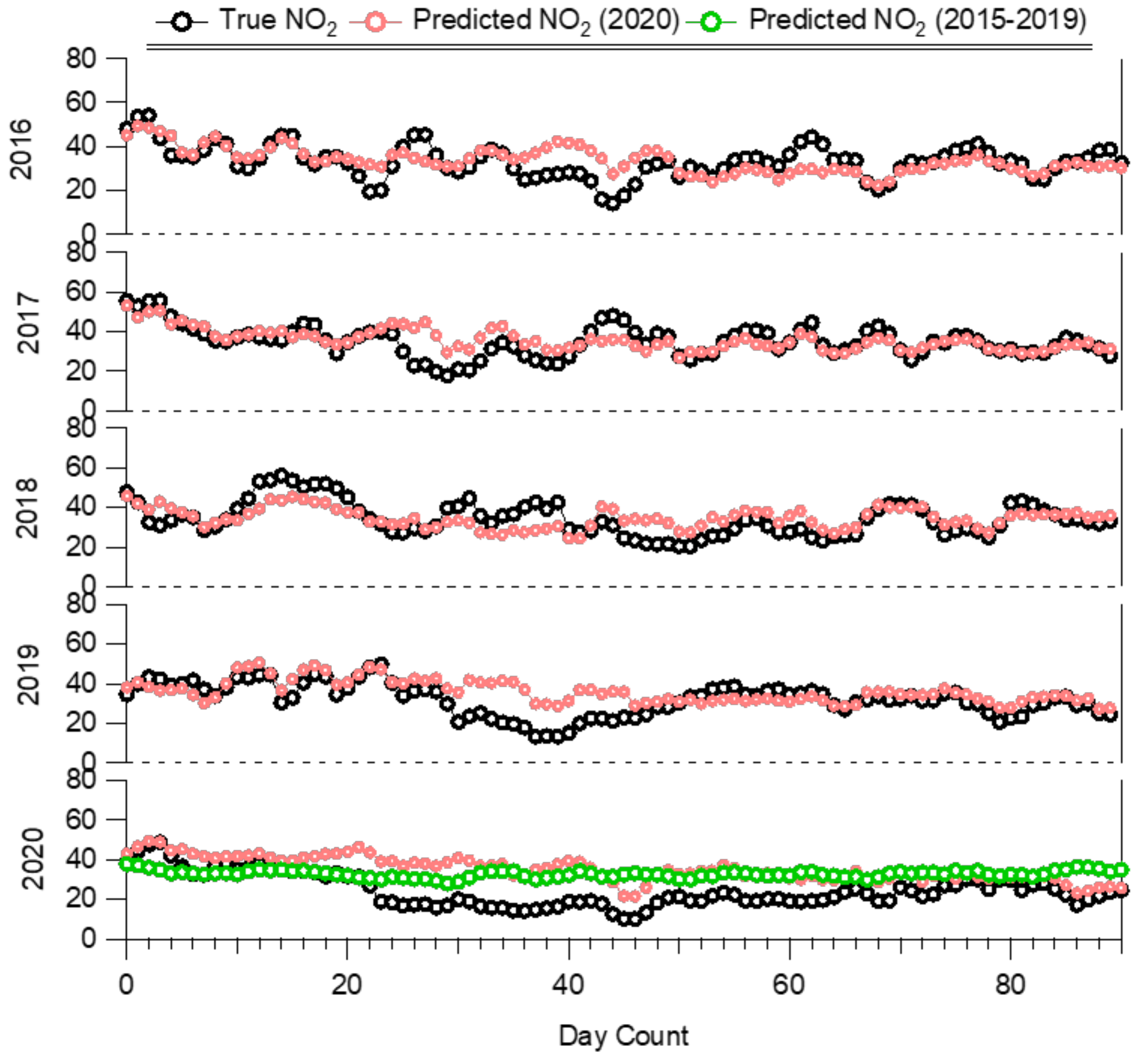

Supplementary Figure 7. Same as in Supplementary Figure 4, but for NO_2.

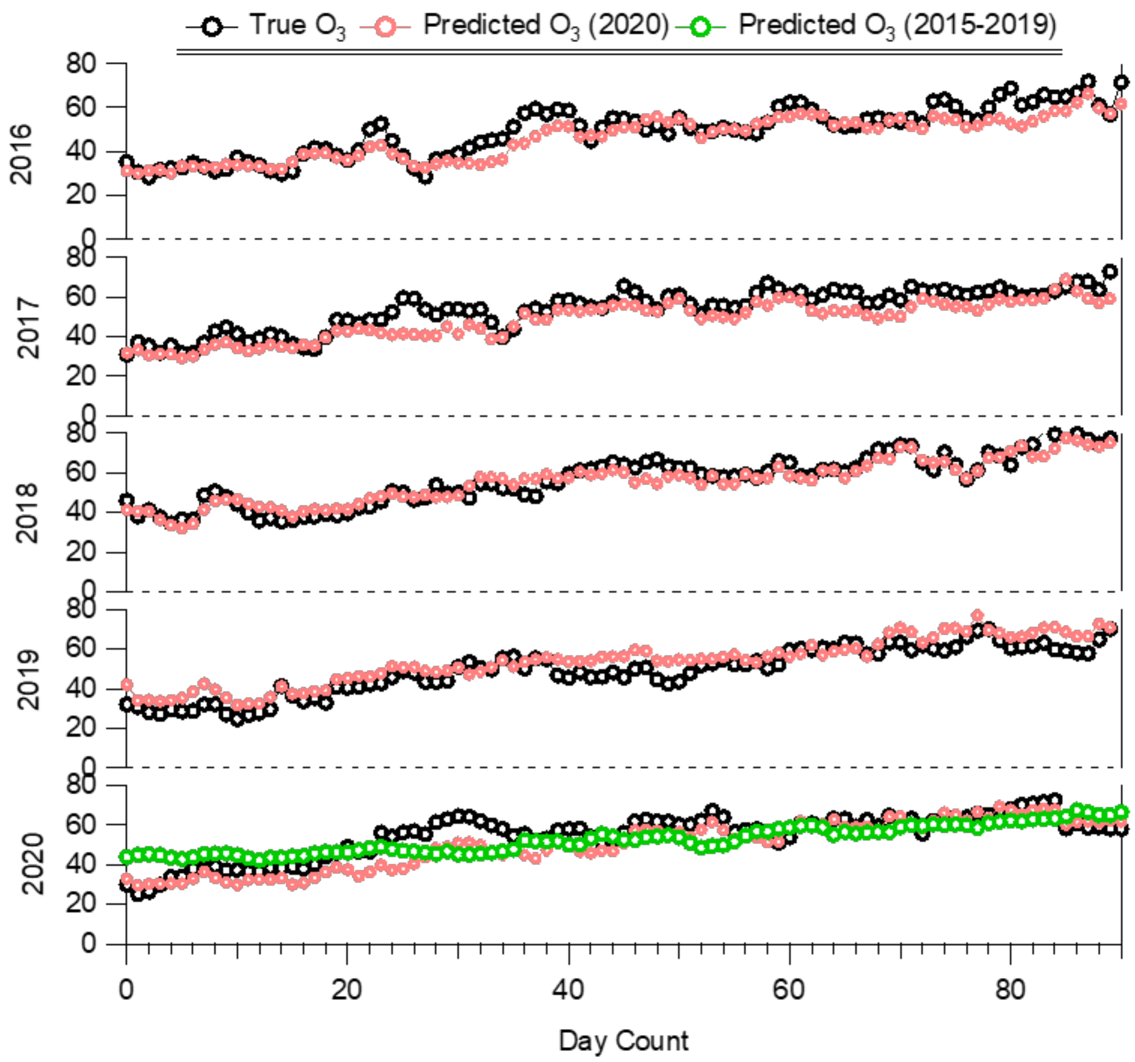

Supplementary Figure 8. Same as in Supplementary Figure 4, but for O_3.

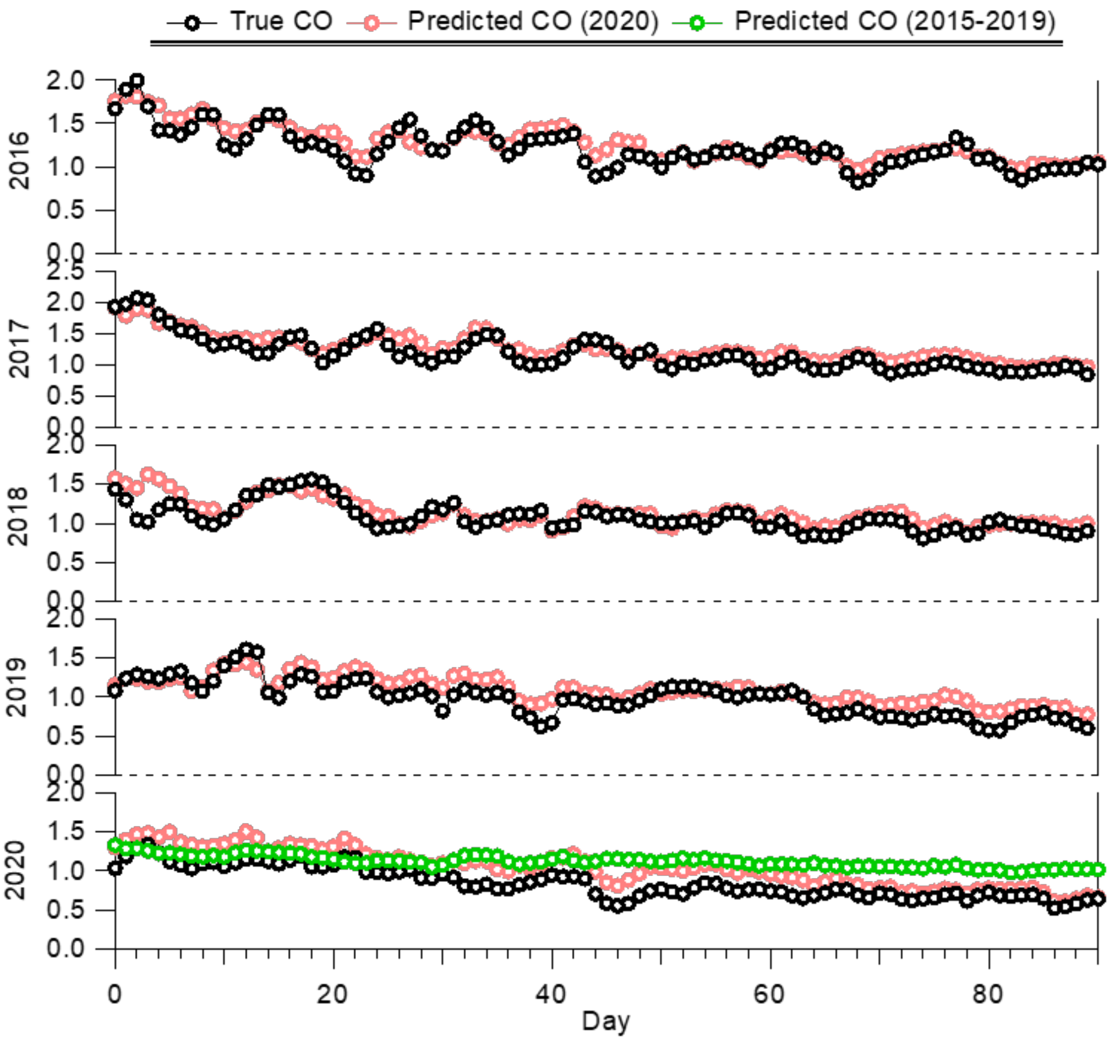

Supplementary Figure 9. Same as in Supplementary Figure 4, but for CO.

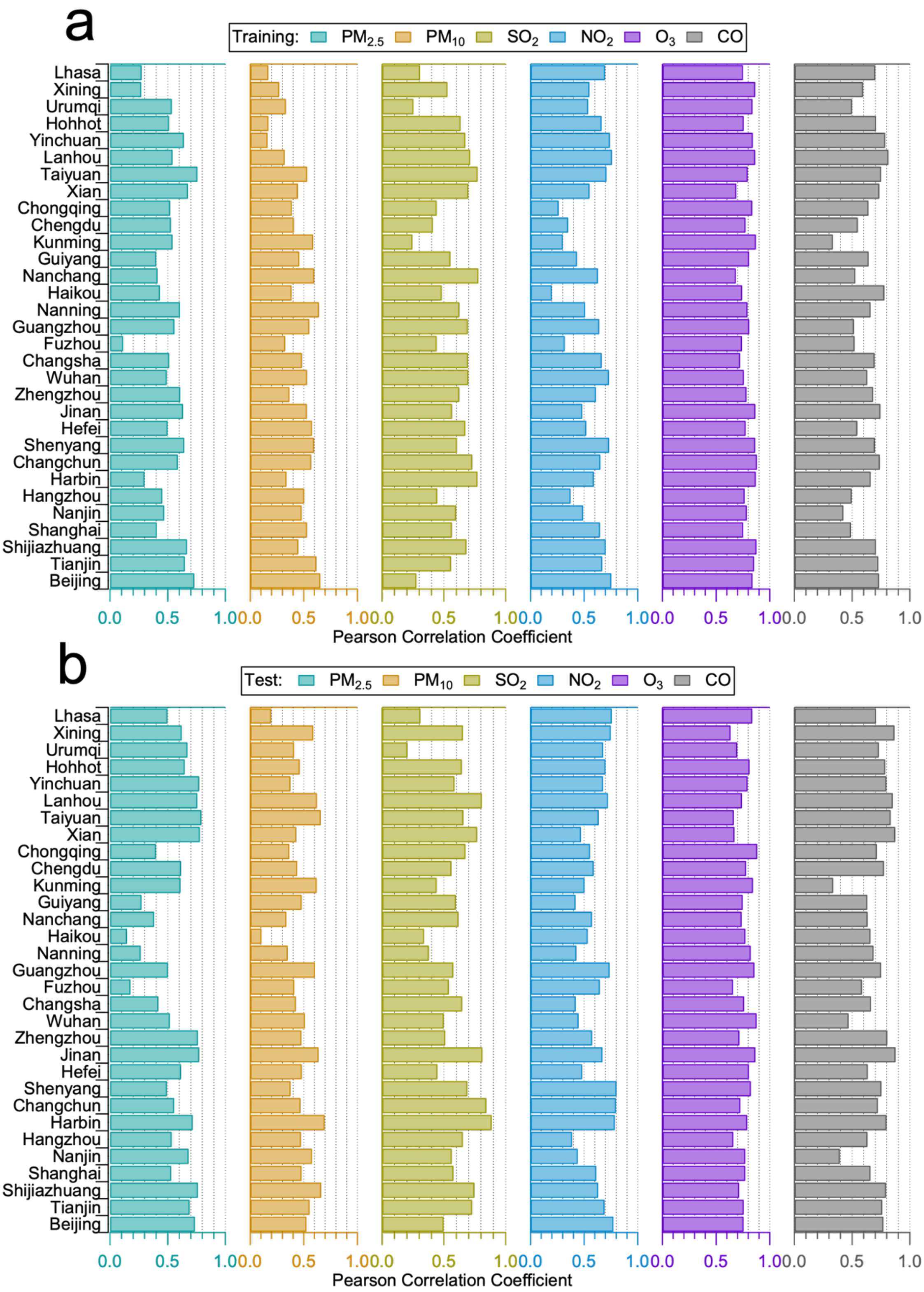

Supplementary Figure 10. The GBM model's performance for each pollutant. The results include training experiments (a) and test experiments (b). The dark green bar represents the Pearson Correlation Coefficient (PCC) of $PM_{2.5}$; the orange bar represents the PCC of PM_{10}; the light orange color represents the PCC of SO_2; the blue bar represents the PCC of NO_2; the purple bar represents the PPC of O_3; the gray bar represents the PCC of CO.

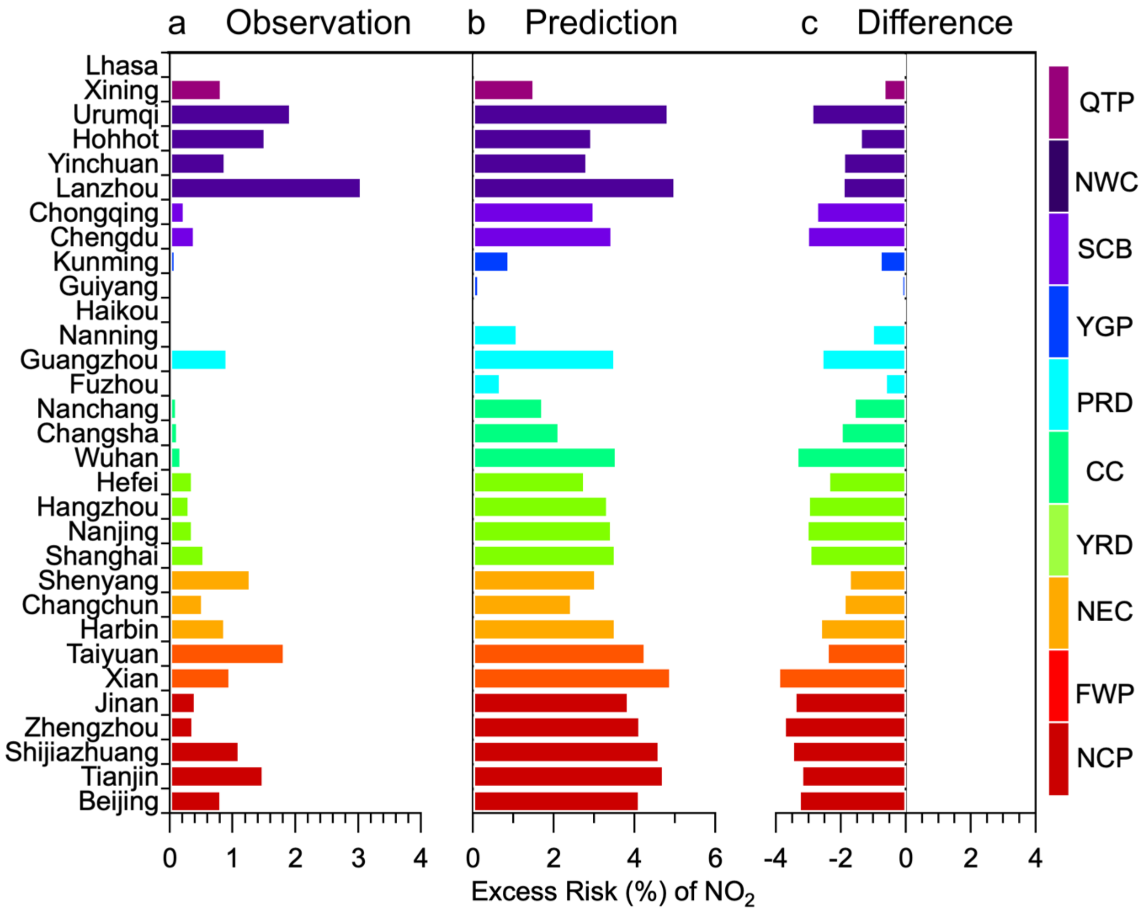

Supplementary Figure 11. Excess Risk (ER) difference (c) of NO_2 between ER of the observed NO_2 (a) and ER of the predicted NO_2 (b) based on the WHO guidelines in 31 capital cities across China during the Total COVID-lockdown period. Different colors in the color bar represent 10 regions (including NCP, FWP, NEC, YRD, CC, PRD, YGP, SCB, NWC, and QTP) clustered by 31 capital cities across China according to their geographic locations.

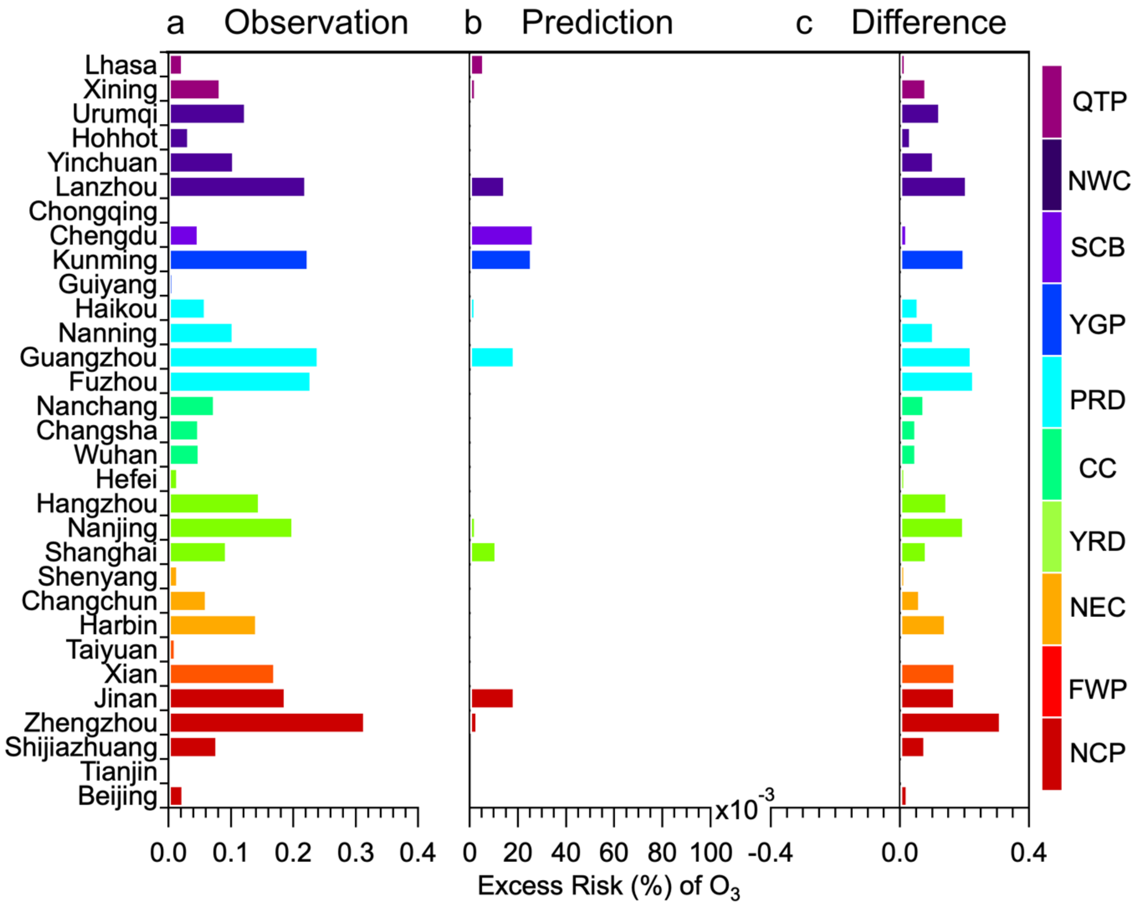

Supplementary Figure 12. Same as in Fig. 11, but for O_3.

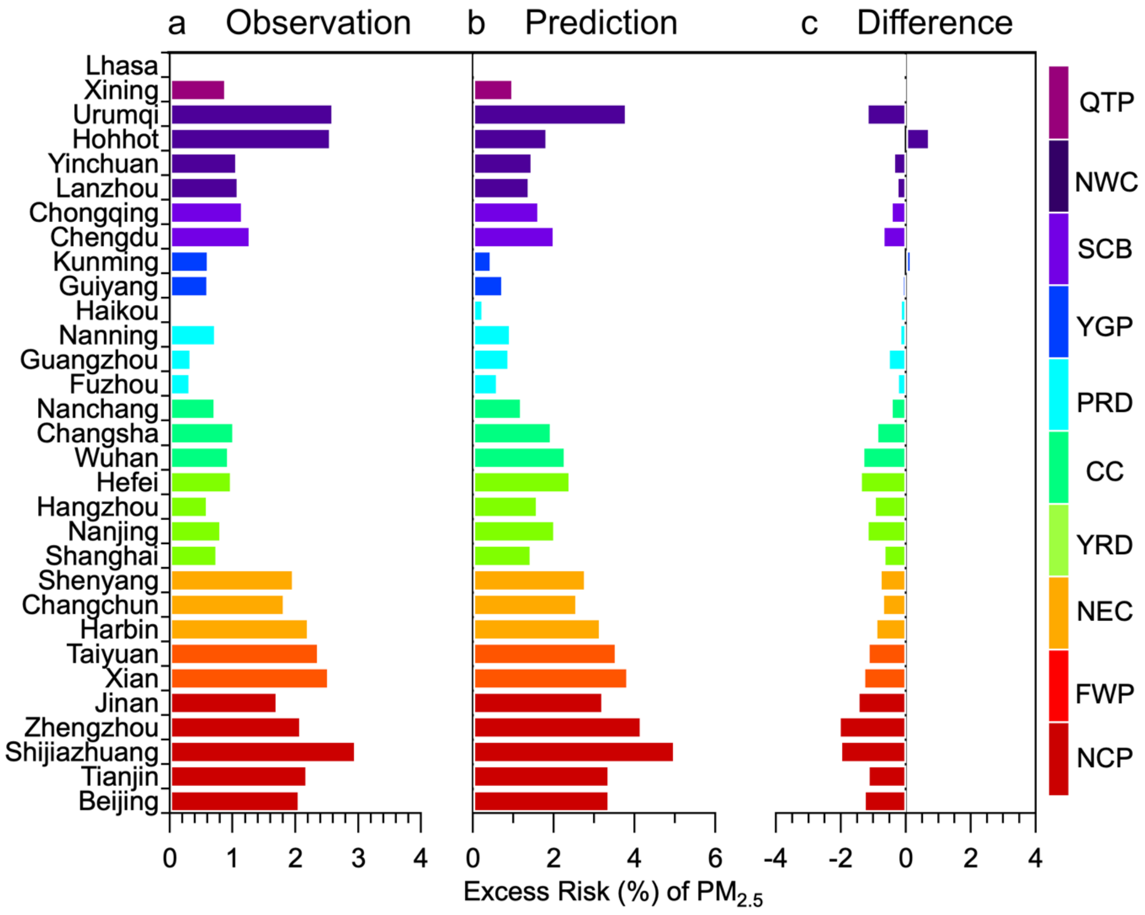

Supplementary Figure 13. Same as in Fig. 11, but for $PM_{2.5}$.

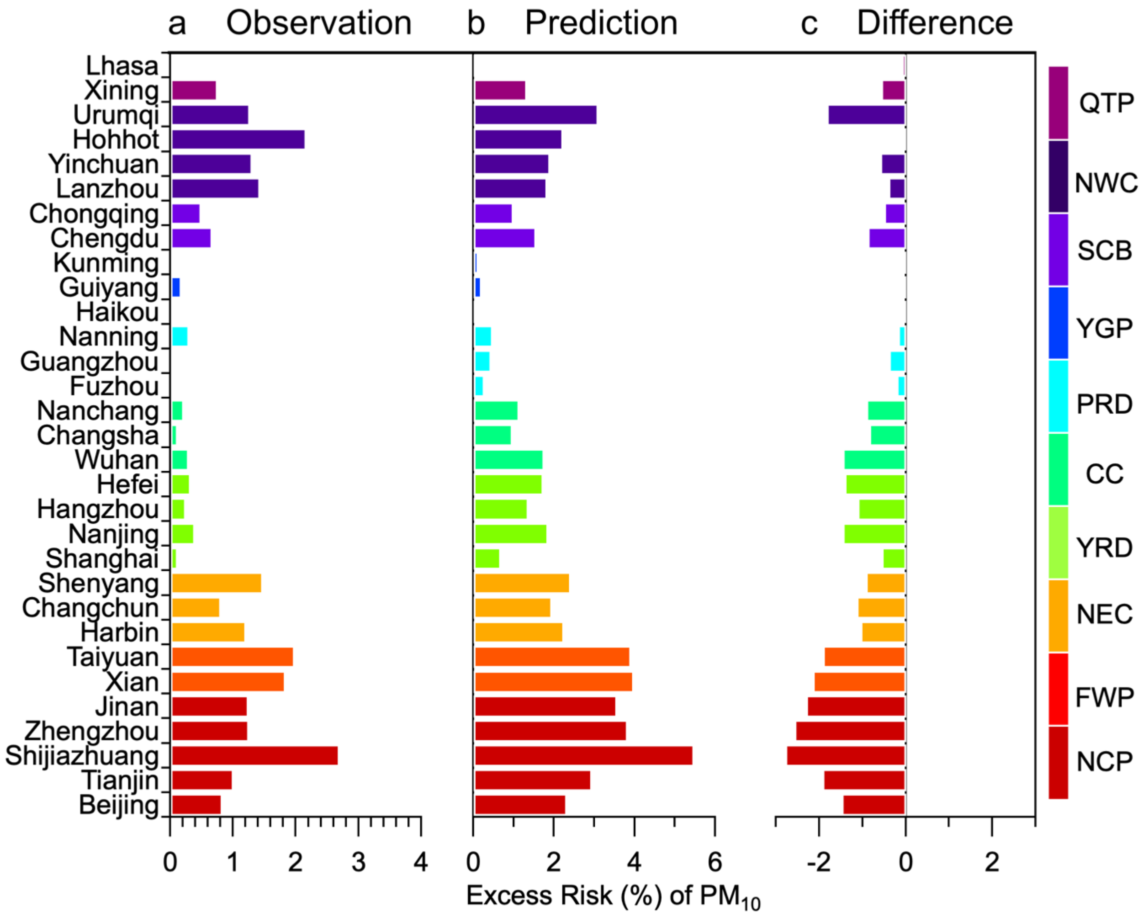

Supplementary Figure 14. Same as in Fig. 11, but for PM_{10}.

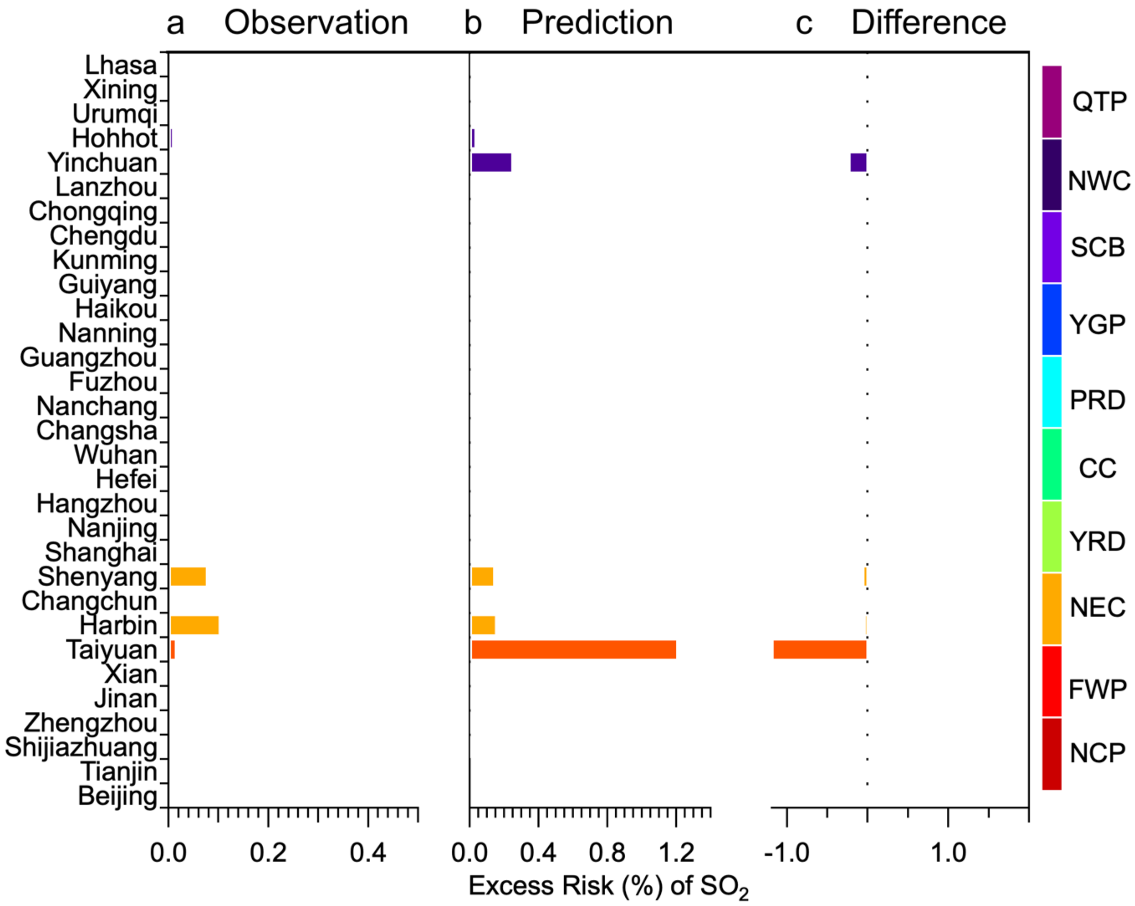

Supplementary Figure 15. Same as in Fig. 11, but for SO_2.

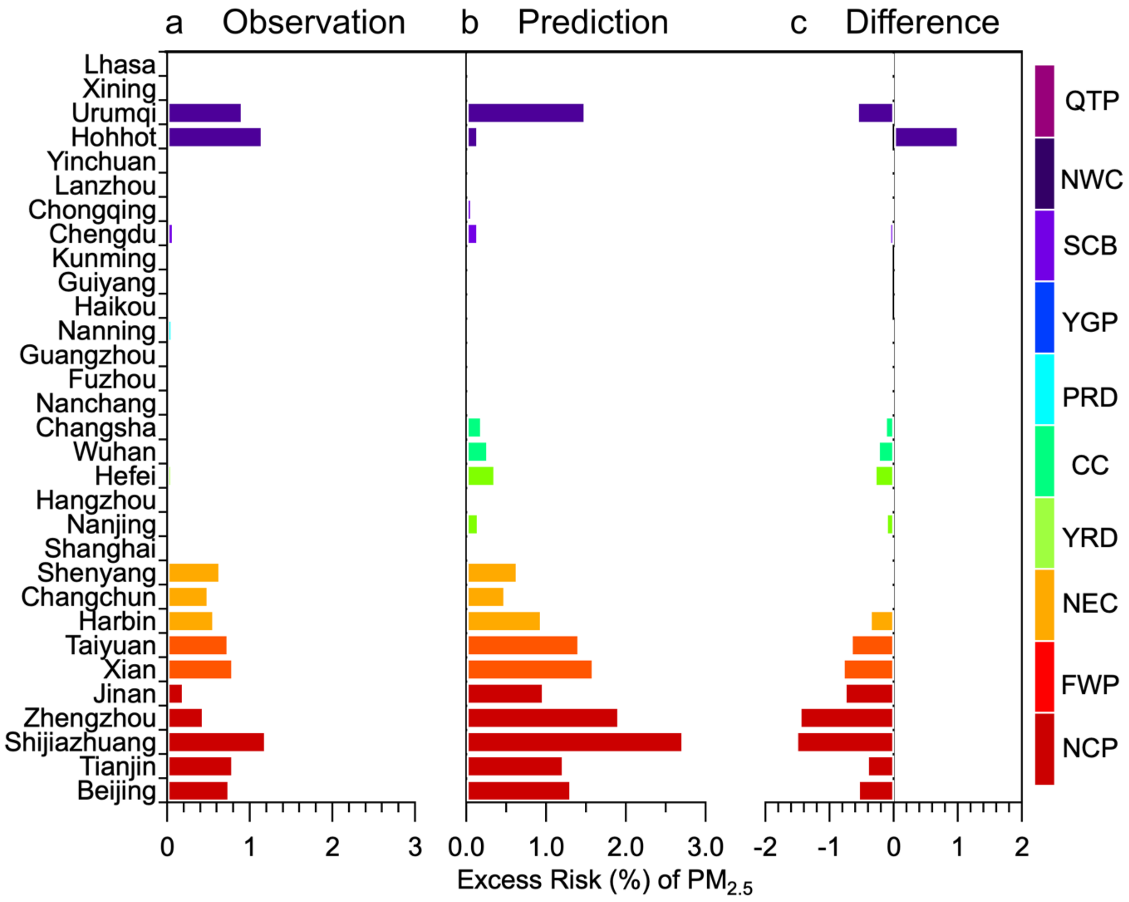

Supplementary Figure 16. Excess Risk (ER) derived from observations and the prediction. Different colors in the color bar represent 10 regions (including NCP, FWP, NEC, YRD, CC, PRD, YGP, SCB, NWC, and QTP) clustered by 31 capital cities across China according to their geographic locations.

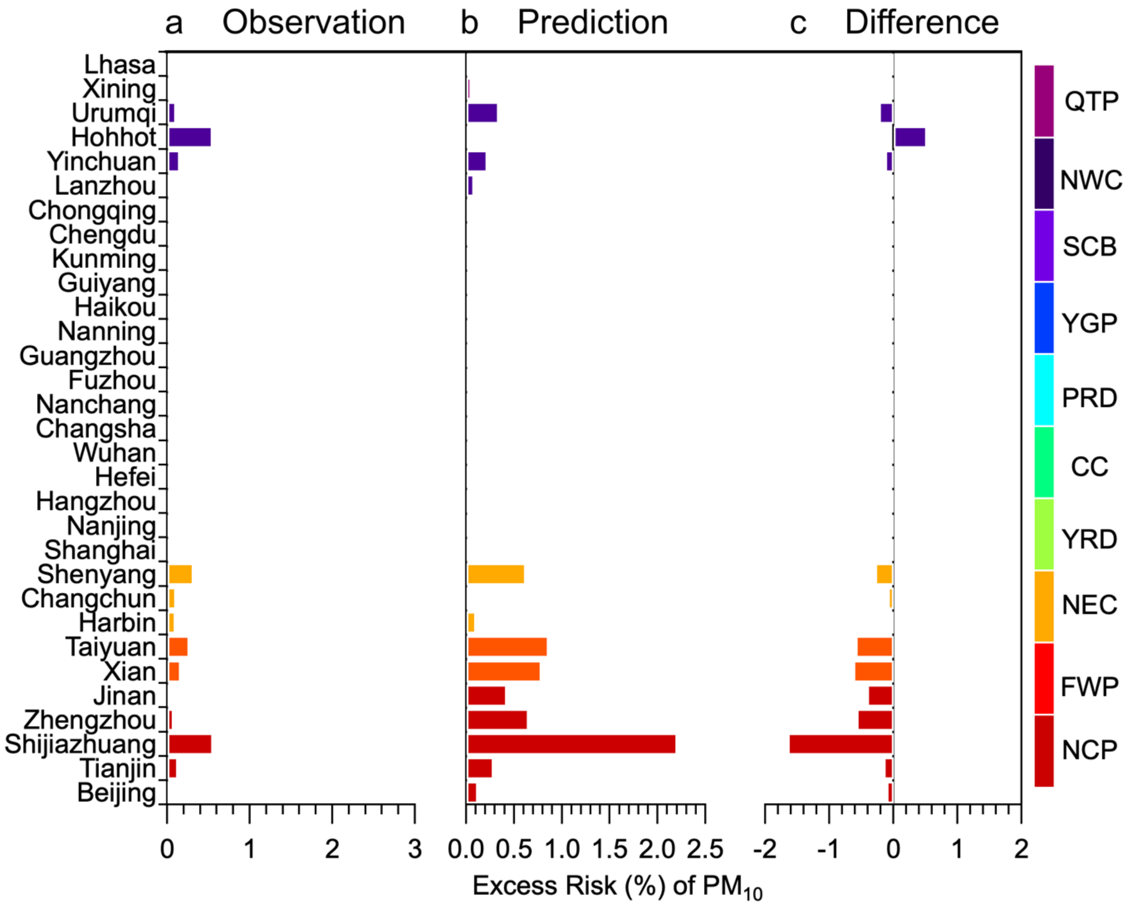

Supplementary Figure 17. Same as in Fig. 16, but for PM_{10}.

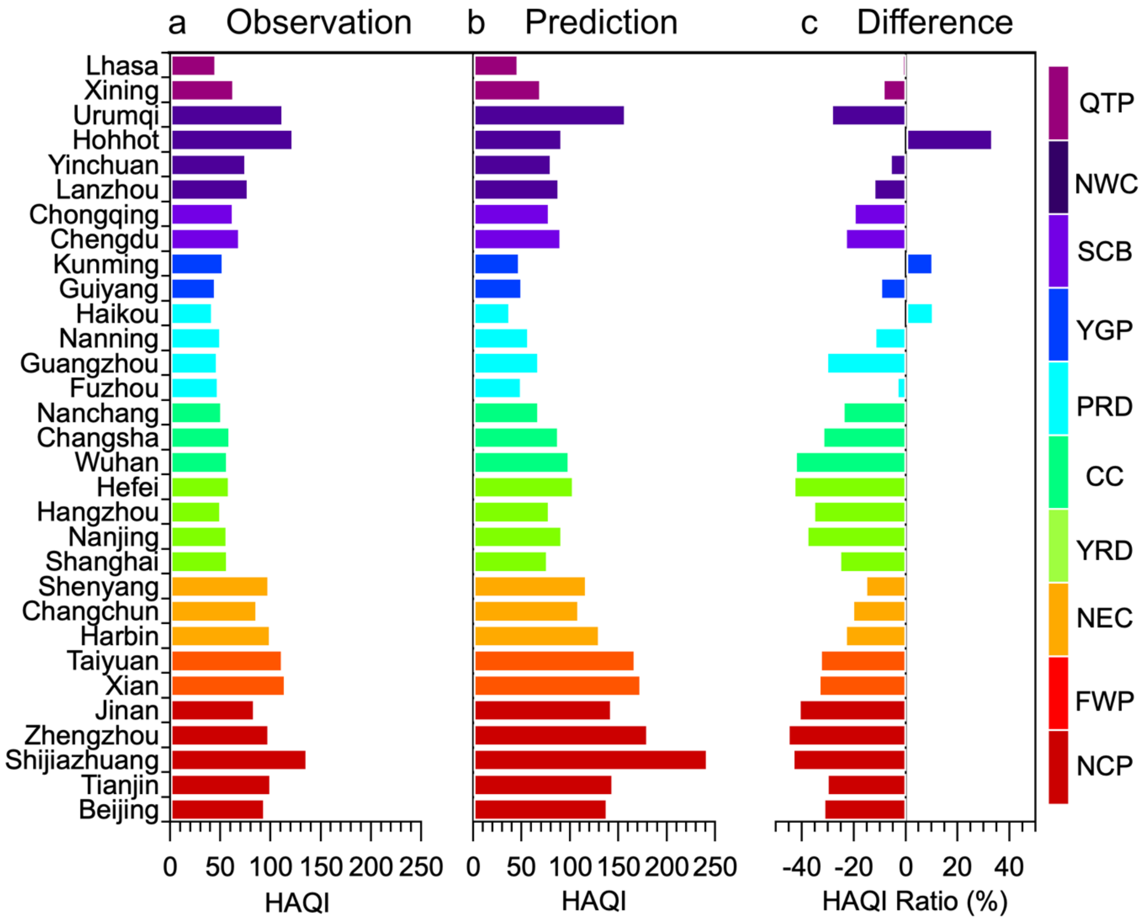

Supplementary Figure 18. **HAQI derived from observations and counterfactual prediction.** Different colors in the color bar represent 10 regions (including NCP, FWP, NEC, YRD, CC, PRD, YGP, SCB, NWC, and QTP) clustered by 31 capital cities across China according to their geographic locations.

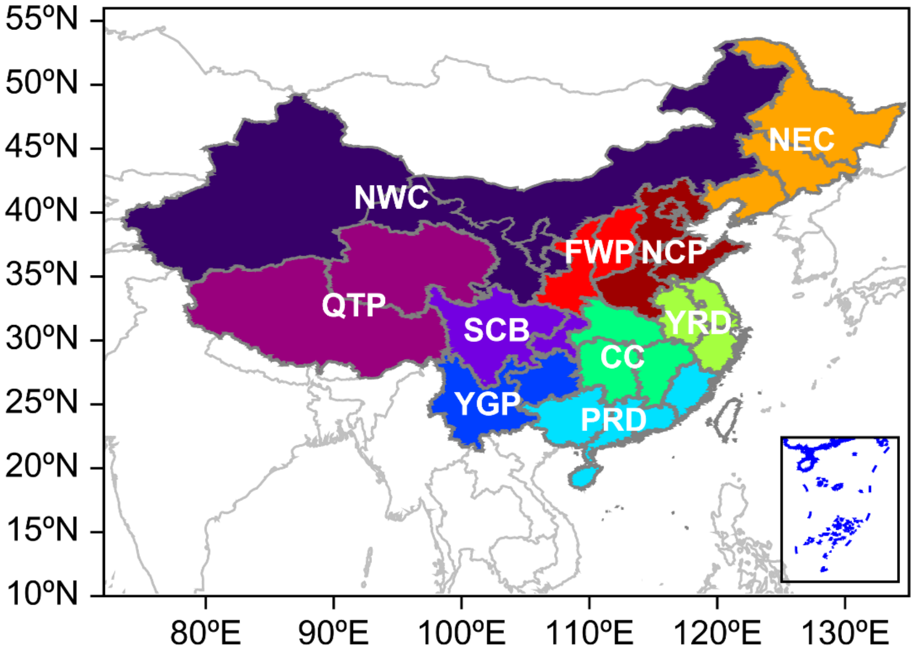

Supplementary Figure 19. The spatial distribution of clustered 10 regions across China. The 10 regions include NWC (Northwest China), NEC (Northeast China), FWP (Fenwei Plain), NCP (North China Plain), YRD (Yangtze River Delta), CC (Central China), SCB (Sichuan Basin), PRD (Pearl River Delta), YGP (Yunnan- Guizhou Plateau), and Qinghai-Tibet Plateau (QTP).

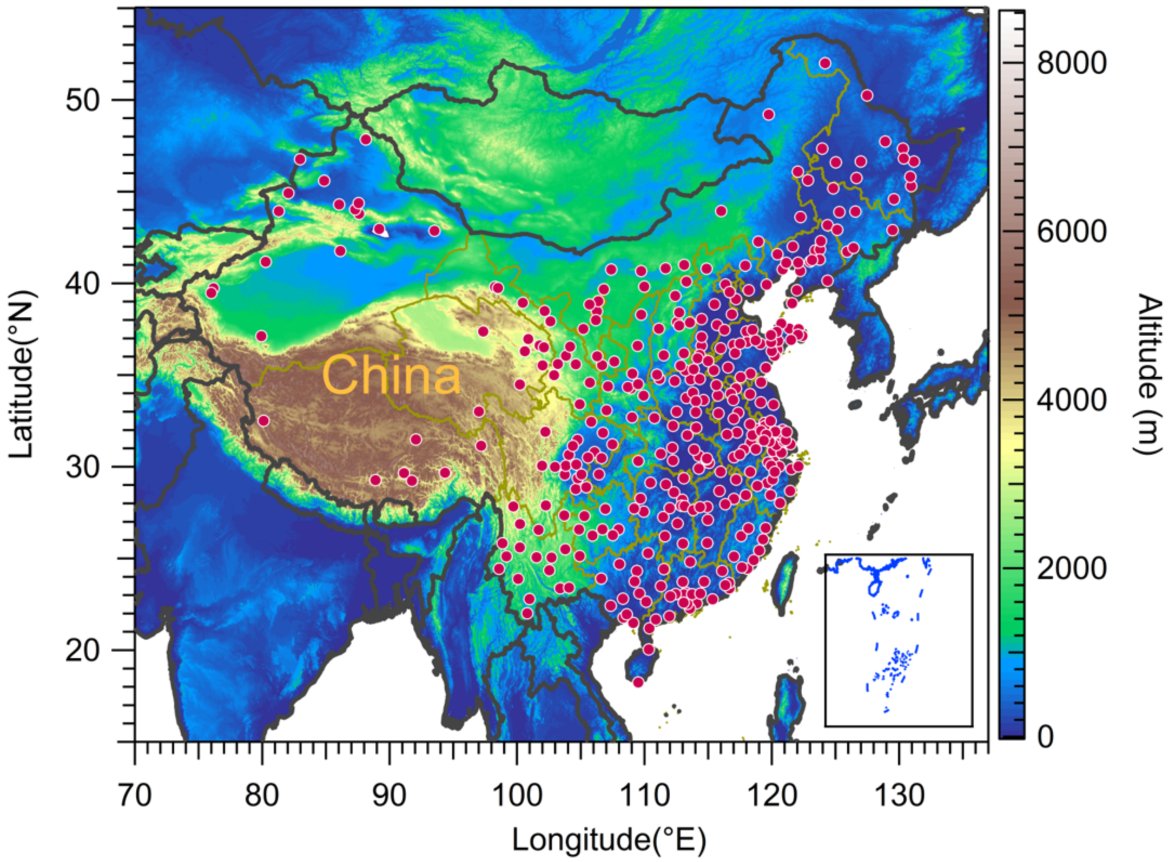

Supplementary Figure 20. The locations of 367 cities in China. The red solid circle represents the location of different cities. The color bar represents the altitude of terrain in China.

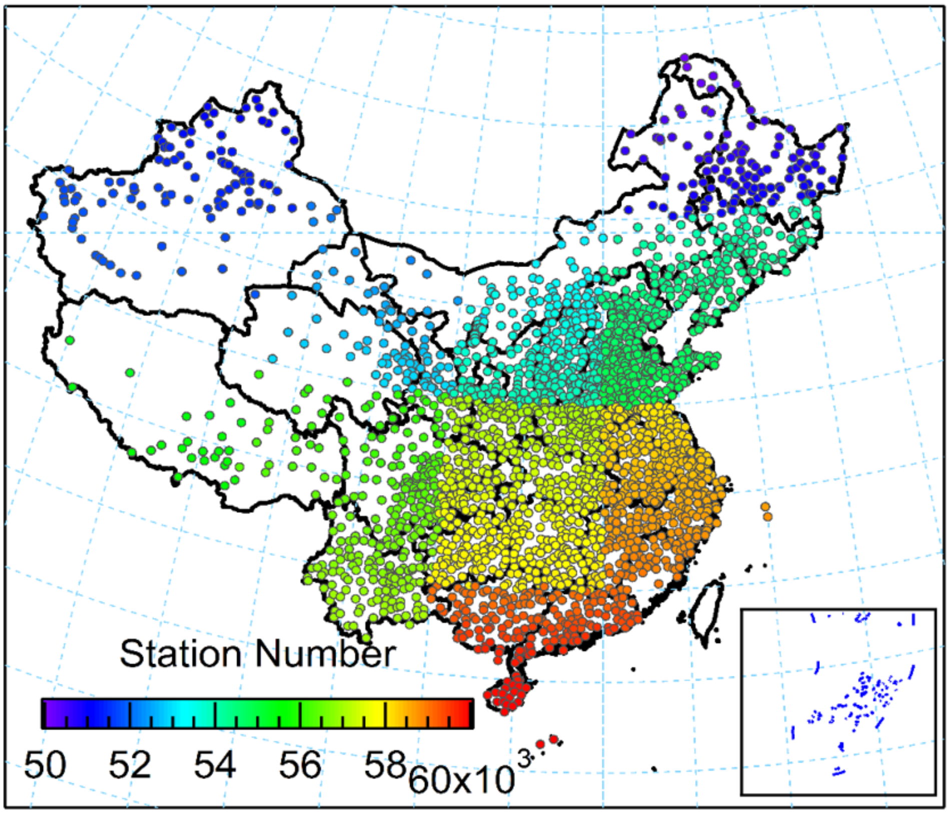

Supplementary Figure 21. The locations of meteorological observation station in China. The color bar represents the station number in China.

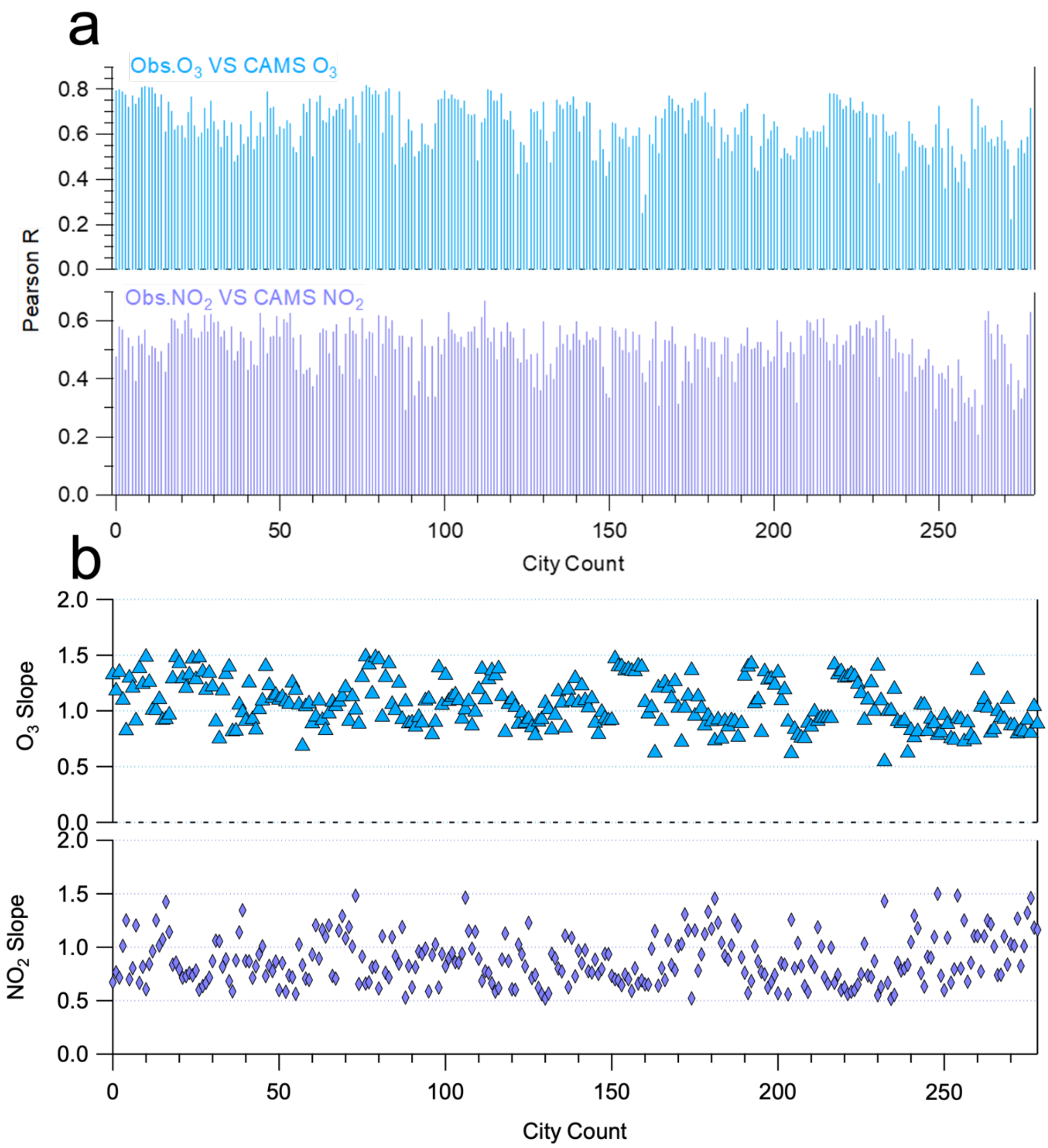

Supplementary Figure 22. Curve fitting results for NO_2 and O_3 in cities across China. The blue and purple bars (a) represent the Pearson Correlation Coefficient (PCC) distribution, and the blue triangle and purple square (b) represent slope distribution for NO_2 and O_3, respectively.

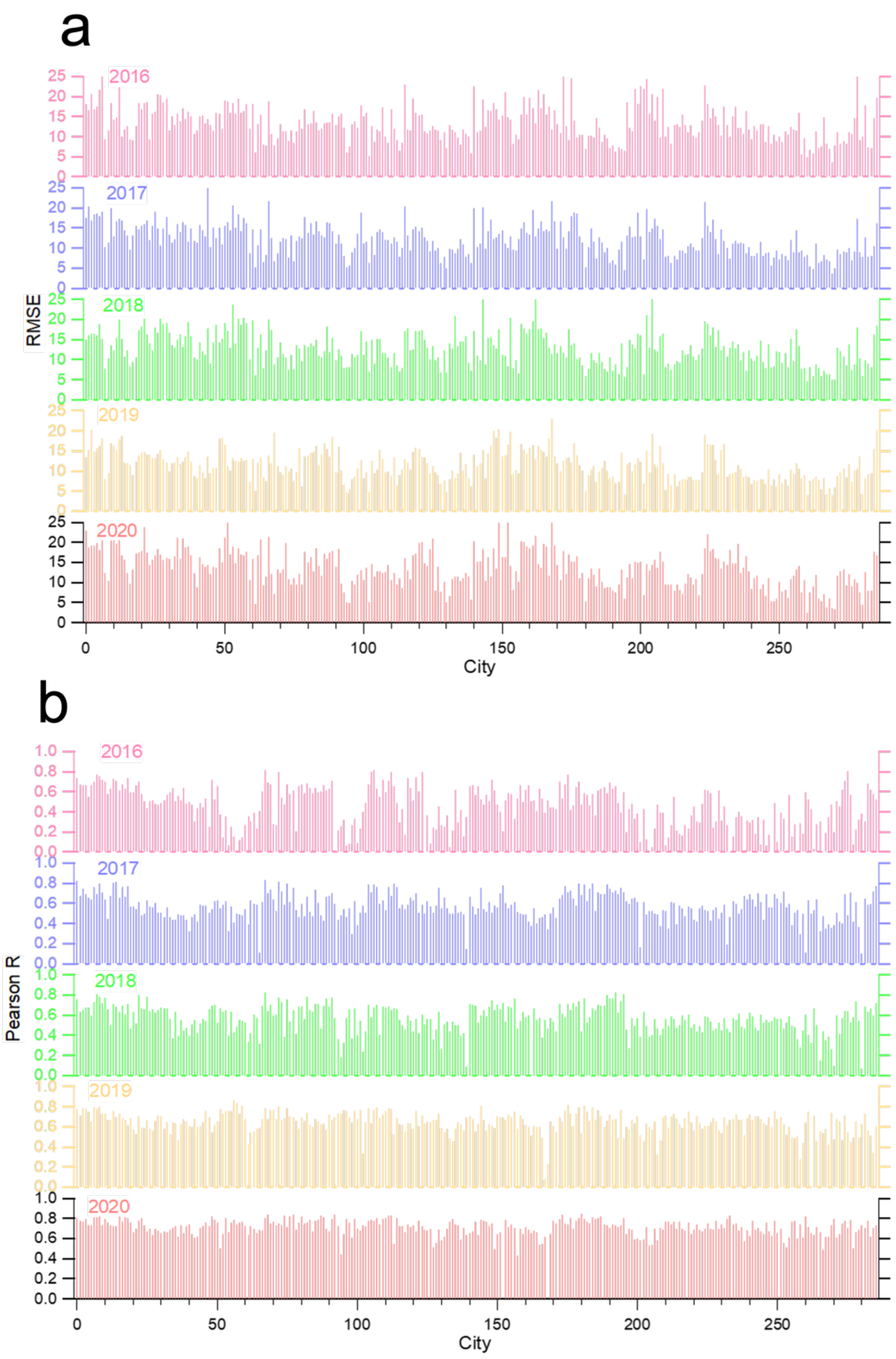

Supplementary Figure 23. The performance for different cross-validation experiments. The top panel represents the PCC distribution of 367 cities across China and the down panel represents the Root Mean Square Error (RMSE) distribution of 367 cities across China. Different color bars represent the PCC and RMSE distribution in different cross-validation experiments including 2016, 2017, 2018, 2019 and 2020.

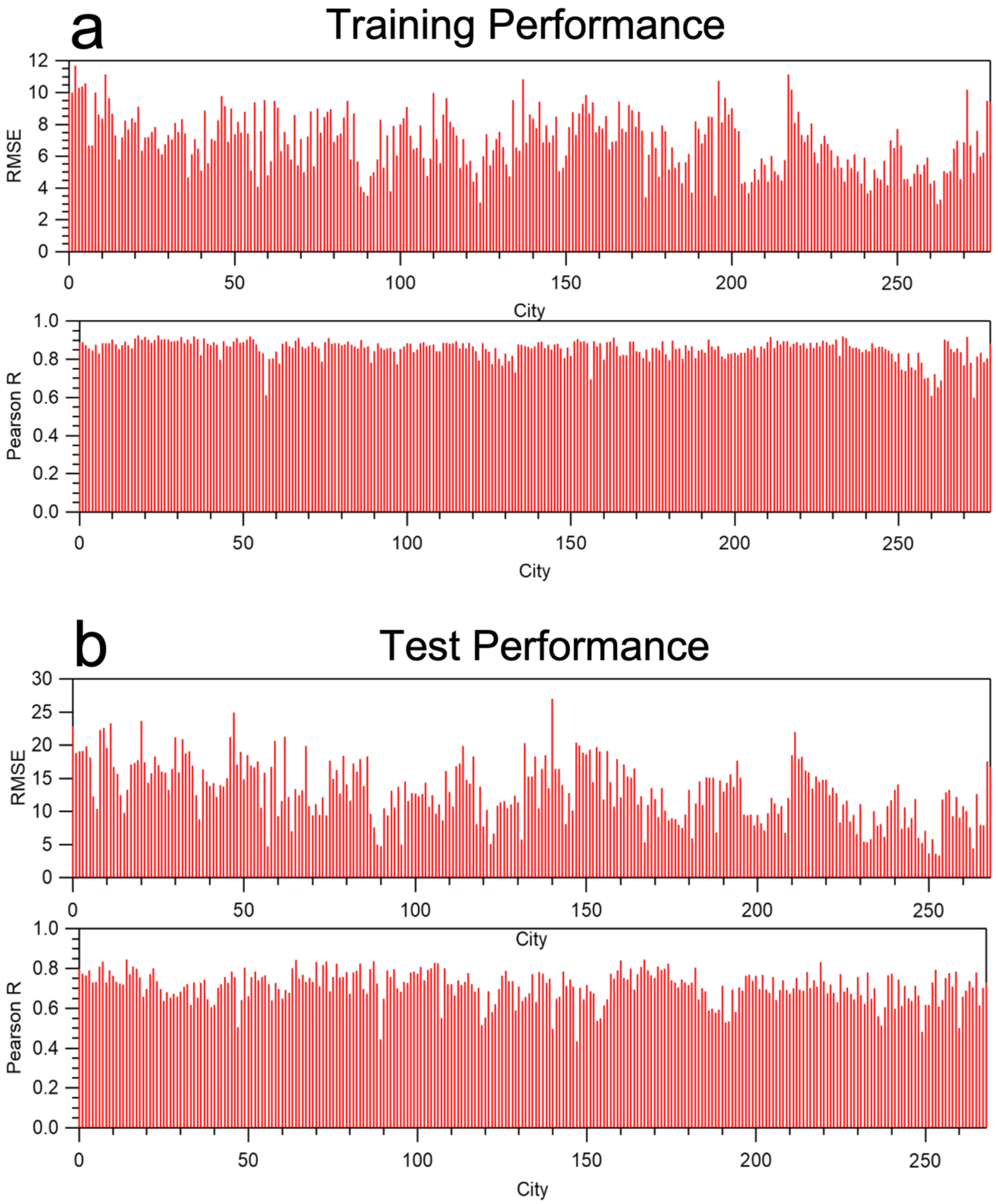

Supplementary Figure 24. The performance of the Machine Learning models. Figure 24a represents the training experiment's PCC and RMSE distribution of 367 cities across China in 2020 and Figure 24b represents the test experiment's PCC and RMSE distribution of 367 cities across China in 2020.

Supplementary Table 1. Definition of different time period

Label	Time Period Definition
Before CNY	days -21 to -8 from the CNY's day
CNY	days -7 to +10 from the CNY's day
Extended COVID-lockdown	days +11 to +42 from the CNY's day (2020)
Total COVID-lockdown*	days -1 to +42 from the CNY's day (2020)

*The Total COVID-lockdown period is from the starting of the COVID-lockdown restrictions to the time when the national average NO_2 concentration back to the normal level.

Supplementary Table 2. Threshold concentrations of pollutants used for health risk.

	WHO Guideline (μg/m^3)	CAAQS Grade II (μg/m^3)
$PM_{2.5}$	15 (24-hour)	75 (24-hour)
PM_{10}	45 (24-hour)	150 (24-hour)
SO_2	40 (24-hour)	150 (24-hour)
NO_2	25 (24-hour)	80 (24-hour)
O_3	100 (8-hour)	160 (8-hour)
CO	4 (24-hour, mg/m^3)	4 (24-hour, mg/m^3)

Supplementary Table 3. Details of capital cities in each region across China.

Regions*	Capital cities*
NCP	Beijing, Tianjin, Shijiazhuang, Zhengzhou, Jinan
FWP	Xi’an, Taiyuan
NEC	Harbin, Changchun, Shenyang
YRD	Shanghai, Nanjing, Hangzhou, Hefei
CC	Wuhan, Changsha, Nanchang
PRD	Fuzhou, Guangzhou, Nanning, Haikou
YGP	Guiyang, Kunming
SCB	Chengdu, Chongqing
NWC	Lanzhou, Yinchuan, Hohhot, Urumqi
QTP	Xining, Lhasa

*It should be noted that the capital cities in each region might have been chosen slightly different from that in other literatures.

Supplementary Table 4. The details of cross validation experiments.

Experiment	Time Period	Time Period for Test	Model Performance
Exp2016	2015.01.01-2015.12.31	2016.01.01-2016.03.31	RMSE=13.4, PCC=0.44
Exp2017	2015.01.01-2016.12.31	2017.01.01-2017.03.31	RMSE=12.1, PCC=0.57
Exp2018	2015.01.01-2017.12.31	2018.01.01-2018.03.31	RMSE=12.7, PCC=0.56
Exp2019	2015.01.01-2018.12.31	2019.01.01-2019.03.31	RMSE=11.4, PCC=0.64
Exp2020	2015.01.01-2019.12.31	2020.01.01-2020.03.31	RMSE=13.5, PCC=0.71
